# Immunogenicity Evaluation of a Rationally Designed Polytope Construct Encoding HLA-A*0201 Restricted Epitopes Derived from *Leishmania major* Related Proteins in HLA-A2/DR1 Transgenic Mice: Steps toward Polytope Vaccine

**DOI:** 10.1371/journal.pone.0108848

**Published:** 2014-10-13

**Authors:** Negar Seyed, Tahereh Taheri, Charline Vauchy, Magalie Dosset, Yann Godet, Ali Eslamifar, Iraj Sharifi, Olivier Adotevi, Christophe Borg, Pierre Simon Rohrlich, Sima Rafati

**Affiliations:** 1 Molecular Immunology and Vaccine Research Lab, Pasteur Institute of Iran, Tehran, Iran; 2 INSERM U1098, Unité Mixte de Recherche, Besançon, France; 3 Etablissement Français du Sang de Bourgogne Franche-Comté, Besançon, France; 4 Université de Franche-Comté, Besançon, France; 5 Department of Electron Microscopy and Clinical Research, Pasteur Institute of Iran, Tehran, Iran; 6 School of Medicine, Leishmaniasis Research Center, Kerman University of Medical Sciences, Kerman, Iran; 7 CHRU de Besançon, Service d′Oncologie, Besançon, France; 8 CHRU de Besançon, Service de pédiatrie, Besançon, France; University of Cape Town, South Africa

## Abstract

**Background:**

There are several reports demonstrating the role of CD8 T cells against *Leishmania* species. Therefore peptide vaccine might represent an effective approach to control the infection. We developed a rational polytope-DNA construct encoding immunogenic HLA-A2 restricted peptides and validated the processing and presentation of encoded epitopes in a preclinical mouse model humanized for the MHC-class-I and II.

**Methods and Findings:**

HLA-A*0201 restricted epitopes from LPG-3, *Lm*STI-1, CPB and CPC along with H-2Kd restricted peptides, were lined-up together as a polytope string in a DNA construct. Polytope string was rationally designed by harnessing advantages of ubiquitin, spacers and HLA-DR restricted Th1 epitope. Endotoxin free pcDNA plasmid expressing the polytope was inoculated into humanized HLA-DRB1*0101/HLA-A*0201 transgenic mice intramuscularly 4 days after Cardiotoxin priming followed by 2 boosters at one week interval. Mice were sacrificed 10 days after the last booster, and splenocytes were subjected to *ex-vivo* and *in-vitro* evaluation of specific IFN-γ production and *in-vitro* cytotoxicity against individual peptides by ELISpot and standard chromium-51(^51^Cr) release assay respectively. 4 H-2Kd and 5 HLA-A*0201 restricted peptides were able to induce specific CD8 T cell responses in BALB/C and HLA-A2/DR1 mice respectively. IFN-γ and cytolytic activity together discriminated LPG-3-P1 as dominant, *Lm*STI-1-P3 and *Lm*STI-1-P6 as subdominant with both cytolytic activity and IFN-γ production, *Lm*STI-1-P4 and LPG-3-P5 as subdominant with only IFN-γ production potential.

**Conclusions:**

Here we described a new DNA-polytope construct for *Leishmania* vaccination encompassing immunogenic HLA-A2 restricted peptides. Immunogenicity evaluation in HLA-transgenic model confirmed CD8 T cell induction with expected affinities and avidities showing almost efficient processing and presentation of the peptides in relevant preclinical model. Further evaluation will determine the efficacy of this polytope construct protecting against infectious challenge of *Leishmania*. Fortunately HLA transgenic mice are promising preclinical models helping to speed up immunogenicity analysis in a human related mouse model.

## Introduction

Cutaneous, Visceral and Mucocutaneous leishmaniasis are three main features of a vector-born parasitic disease caused by *Leishmania* genus and transmitted by sandfly bite [1]. Leishmaniasis can be transmitted in many tropical and subtropical countries, and is found in parts of about 98 countries on 5 continents. Different forms of the disease predominate in different regions of the world. Countries like Morocco, Nepal, India, China, Iraq and Bangeladesh are mostly involved with visceral leishmaniasis while others like Algeria, Syria, Iran, Tunisia, Afghanistan, Pakistan and Saudi Arabia are involved with cutaneous form. Brazil, is almost exclusively involved with all three forms of the disease at a very high incidence rate [2].

Current control relies on chemotherapy to alleviate the disease and on vector control to reduce transmission. A few drugs are available for chemotherapy but facing problems such as high toxicity, variable efficacy, inconvenient treatment schedules, costs and drug resistance [3]. Vector control has also appeared extremely difficult due to sand fly generalization and adaption to many different micro-landscapes [4]. Thus an effective vaccination would be of great interest to control this expanding disease. Unfortunately despite all efforts made using different vaccination strategies [5], [6], [7], no protective vaccine for human is available to control the disease except for a multi-protein vaccine namely LEISH-F(F1, F2, F3) which is still in clinical trial and has not entered the market yet [8], [9], [10].

*Leishmania* is an obligatory intracellular parasite residing and proliferating inside macrophages as ultimate host cells. Therefore with no doubt IFN**-γ** plays a vital role in controlling the infection since it induces the signal for nitric oxide production by macrophages. Nitric oxide is a nitrogen metabolite that inhibits parasite survival [11], [12]. Consensually CD4^+^ Th1 cells have been considered the main IFN**-γ** providers in *Leishmania* specific response, but today's knowledge also remarks the CD8^+^ cytotoxic T cells (Tc1) role in this scenario [13], [14], especially in controlling secondary *Leishmania (L.) major* infection. [15], [16], [17]. There was an unresolved paradigm around the role of these cells controlling primary infection [18], [19], [20] but Belkaid's elegant experiment with low rather than high dose inoculation finally shed light on this enigma. Intradermal low-dose (100–1000) metacyclic challenge with *L. major* (resembling the natural infection transmitted by sandfly bite) in C57BL/6 mice depleted of CD8^+^ T cells successfully established a progressive infection defeating the immune system [21]. Later on, Uzonna *et al.* delineated a transient Th2 response at early stages of low dose challenge that was modified and diverted to Th1 only in the presence of IFN**-γ** producing CD8^+^ T cells and not in CD8^+^ T cell depleted mice [22].

Besides their IFN-γ production [23], cytolytic activity of CD8^+^ T cells has also been under question [24], [25], [26], [27], [28]. On one hand the massive proliferation of the parasite in non-ulcerative nodules from patients suffering from diffuse cutaneous leishmaniasis and post Kala-Azar dermal leishmaniasis has been ascribed to CD8^+^ T cell exhaustion due to long lasting infection [29], [30]. On the other hand, the parasite-free pathologic lesions of patients suffering from mucosal leishmaniasis have been ascribed to hyperactivity of CD8^+^ T cells at involved tissue [31], [32]. Whether the cytolytic activity is responsible for parasite eradication directly by apoptosis or indirectly by disrupting parasite infected macrophages is unclear.

Besides all other vaccination strategies, today protective and therapeutic peptide-based vaccine concept has drawn attraction in the field of intracellular infections [33], [34], [35] and cancer [36], [37] where multi-CD8 cytotoxic T cell responses are crucial mediators of immunity. Since the evidence continues to pile up about CD8^+^ T cells role [38], [39], [40], [41], [42], peptide vaccine might open a new way in the battle over leishmaniasis.

In our previous study six known proteins from *L. major* were screened for best HLA-A2 binding 9 mer peptides by immunoinformatics tools. A few peptides from *L. major* Stress Inducible Protein-1 (*Lm*STI-1) and Lipophosphoglycan Protein-3 (LPG-3) were then shown to be immunogenic stimulating PBMC from cutaneous leishmaniasis recovered individuals [43]. Since DNA constructs are well known for CD8^+^ T cell stimulation, we rationally designed a DNA construct encoding previously selected peptides to evaluate the processing, presentation and immunogenicity of various encoded epitopes in a preclinical mouse model humanized for HLA class I and II molecules (HLA-DRB1*0101/HLA-A*0201). These preclinical models are precious tools in hand to study the immunogenicity of *in-silico* selected peptides for vaccination purposes in humans.

## Material and Methods

### Ethics statement

Transgenic animals, homozygous for all modified genetic characters, were bred at the IBCT animal facility of INSERM UMR1098, Besançon, France (authorization number D-25-056-7). The protocol including maintenance, anesthesia (under standard isoflurane inhalation) and euthanasia (via cervical dislocation) was reviewed and approved by the competent authority at INSERM UMR1098 for compliance with the French and European Regulations on Animal Welfare (protocol number 10005).

### Design of the polytope construct

HLA-A2 restricted epitopes from 4 different *Leishmania* proteins (CPB and CPC, 5 peptides, *Lm*STI-1, 4 peptides and LPG-3, 4 peptides) along with 4 epitopes presented in H-2Kd allele context from BALB/c mice were included. Peptides were lined up together to satisfy two basic criteria: least junctional peptides (neoepitope, generated by the juxtaposition of two authentic epitopes) and most cleaved peptides of interest out of the polytope sequence. To this end all possible arrangements (24 different arrangements) without spacer and with 4 different spacers: AAA (3 alanine residues) [44], [45], AAY (2 alanine and one tyrosine residues) [46], [47], K (lysine) [48], [49] and AD (one alanine and one aspartic acid residues) [50] (a total of 120 different arrangements for each protein) were analysed with two common on-line algorithms for proteasome cleavage: NetCTL 1.2 [51] and nHLApred [52]. This approach was followed for peptide arrangements of each individual protein and then the most preferable arrangements satisfying both basic criteria were selected to be combined again in a longer polytope. Using NetCTL 1.2 a few combinations were selected. To finalise the selection process we checked for the hydrophobicity pattern of the different combinations. Final arrangement flanking with additional sequences as, *Hin*dIII restriction site, kozak sequence and mouse ubiquitin sequence at amino-terminal (N-terminal) and Tetanus Toxoid universal Th1 epitope (TT_830_) with *Bam*HI restriction site at carboxy-terminal (C-terminal) was synthesized by BIOMATIK company (Canada). 921 base pair (bp) long sequence (PT) was codon optimized for optimal expression in mouse cells and was received as pUC57-PT (Codon Optimization was done using BIOMATIK proprietary software. 15% cut off was used for codon efficiency and any codon below 15% was removed except for positions with strong secondary structures. Secondary structure was checked using a build in M-fold module (Internal ribosomal binding sites were removed). Table 1 summarizes the HLA restriction and immunoinformatics characteristics of individual peptides in the construct (6 HLA-A2 restricted and 4 H-2Kd restricted peptides marked in bold were analysed in this study).

**Table 1:** Characteristics of *in silico* predicted *L. major* specific CD8^+^ T cell 9-mer peptides included in the polytope construct.

			Scores predicted by online immunoinformatics software						
Protein (GeneDB accession number)	Peptide sequence	HLA Restriction	SYFPEITHI (1)	BIMAS (2)	EpiJen (3)#	RANKpep^/^Proteasome cleavage(4)	nHLAPred (5)	NetCTL (6)	Multipred (7)
CPB (LmjF08.1080)	LMLQAFEWV	**HLA-A** * **0201**	22	1617	+	72/−	1	1.255	MB^a^
	QLNHGVLLV		28	159	+	73/+	1	1.055	MB
	LLTGYPVSV		28	118	+	91/−	1	1.284	MB
CPC (LmjF29.0820)	FLGGHAVKL		27	98	+	73/+	0.97	1.097	MB
	LLATTVSGL		29	83	+	90/+	1	1,137	MB
*Lm*STI-1 (LmjF08.1110)	**LLMLQPDYV** * **(P4)**		23	1179	+	68/+	1	1.027	MB
	**ALQAYDEGL (P6)**		24	10	+	63/+	0.93	1.218	MB
	**QLDEQNSVL (P3)**		22	14	+	64/+	1	0.791	MB
	**YMEDQRFAL (P2)**		21	108	+	72/+	0.99	1,102	MB
LPG-3 (LmjF29.0760)	**LLLLGSVTV (P1)**		30	437	+	86/+	1	1.023	MB
	FLVGDRVRV		25	319	+	80/+	1	1.191	MB
	**MLDILVNSL (P5)**		28	33	+	76/+	1	1.142	MB
	MTAERVLEV		25	15	+	74/+	1	1.181	HB^b^
LmjF25.0150	**AYSVSASSL (Kd1)**	**H-2Kd**	28	2880	-	-	-	-	-
LmjF14.0650	**SYETGSSTL (Kd2)**		25	2400	-	-	-	-	-
LmjF29.2650	**FYQEAAELL (Kd3)**		27	2400	-	-	-	-	-
LmjF29.0867	**SYSSLVSAL (Kd4)**		28	2880	-	-	-	-	-

* Peptides analyzed in HLA transgenic mice (P1–P6) and BALB/c mice (Kd1–Kd4) are in bold.

a Moderate binder.

b High binder.

# Peptide falling over pre-set threshold.

(1) http://www.syfpeithi.de/bin/MHCServer.dll/EpitopePrediction.htm.

(2) http://www-bimas.cit.nih.gov/molbio/hla_bind/.

(3) http://www.jenner.ac.uk/EpiJen/.

(4) http://immunax.dfci.harvard.edu/Tools/rankpep.html.

(5) http://www.imtech.res.in/raghava/nhlapred/comp.html.

(6) http://www.cbs.dtu.dk/services/NetCTL/.

(7) http://antigen.i2r.a-star.edu.sg/multipred.

CPB (Cathepsin L-like Protease or Type I Cysteine Proteinase), CPC (Cathepsin L-like Protease or Type I Cysteine Proteinase), *Lm*sTI-1 (*L.major* Stress Indussible Protein), LPG-3 (Lipophosphoglycan Biosynthetic Protein).

### Cloning pathway

To meet the objectives of this study, pUC57-PT (BIOMATIK, Canada) was subject to a few cloning steps. 918 bp long fragment digested by *Hin*dIII – *Bam*HI (Roche, Germany) enzymatic reaction was directly cloned into pEGFP-N3 plasmid (Clontech, USA) digested with the same enzymes. Polytope sequence was inserted in-frame upstream to the EGFP sequence generating pEGFP-PT. The polytope sequence in tandem with EGFP was digested out of the pEGFP-PT by *Bgl* II – *Not* I restriction enzymes (Roche, Germany) and sub-cloned into pLEXSY-neo-2 plasmid (Jena Biosciences, Germany) making pLEXSY-PT-EGFP. Eventually polytope sequence was amplified with a set of primers (forward: CGCAAGCTTACCATGCAGATTTTCG and Reverse: AATGGATCC **CTA**CACCAGCAGCACGCC, MWG, Germany) with *Hin*dIII and *Bam*HI restriction sites (underlined) and stop codon on reverse primer (bold). The PCR product was directly cloned into pcDNA3.1(+) vector (Invitrogen, USA) which was digested with *Hin*dIII and *Bam*HI. Recombinant pcDNA-PT was next subject to sequencing.

### Cell lines

African green monkey kidney fibroblast-like cell line, COS-7 (ATCC CRL-1651), was cultured in RPMI-1640 medium (Sigma, Germany) supplemented with 10% inactivated fetal calf serum (Gibco, USA), 2 mM L-Glutamine, 10 mM HEPES and 50 µg/ml Gentamicin (all from Sigma, Germany). CT26, an undifferentiated colon carcinoma cell line from BALB/c origin (ATCC CRL-2638) was cultured in DMEM – Glutamax (+) medium supplemented with 1% penicillin/streptomycin antibiotic mixture and 10% inactivated fetal bovine serum (All from Gibco, USA). RMA/s cells, Transporter associated with antigen processing (TAP) deficient cells from C57BL/6 origin transfected with HLA-A*0201 were cultured in RPMI-1640 Glutamax (+) medium supplemented with 1% penicillin/streptomycin antibiotic mixture and 10% inactivated fetal bovine serum (FBS).

### HLA-Transgenic mice

The humanized HLA-DRB1*0101/HLA-A*0201 were kindly provided by F.A. Lemonier, Pasteur Institute of Paris and were bred in animal facility of INSERM UMR1098. These mice are double knockouts of H-2 class I and class II genes and thus express only human MHC molecules (H-2 class I (*β*2m^O^)/class II (IA *β* ^O^)-KO). These C57BL/6 background mice express human alpha-1/alpha-2 domains of HLA-A*0201 heavy chain and murine alpha-3 domain covalently linked to human beta-2 microglobulin domain.

### *In-vitro* evaluation of polytope expression using COS-7 cells

Recombinant pEFGP-PT was purified (Qiagen midi-plasmid purification kit, Germany) and transiently transfected into COS-7 cells by means of linear Polyethylenimine 25 KDa (LINPEI.25-Polysciences, USA), as previously described [53]. Briefly, freshly prepared DNA/PEI complex in HBS buffer with 18 µl of PEI at NrE (Number of Equivalents)  = 7 and 5 µg of plasmid DNA was mixed with COS-7 cells (5×10^4^ cell/well in opti-MEM serum free medium (Invitrogen, USA)) at 70% confluency. In this experiment pEGFP-N3 plasmid without insert was used as control. Six hours later, cells were washed and incubated for further 18 hours. Green fluorescence of GFP expressing cells was detected both by fluorescent microscope (Nikon-Japan) and by flow cytometry (Becton Dickinson FACScalibur, USA) after 24 hours.

### Stable transfection of *Leishmania tarentolae* with pLEXSY-PT-EGFP

Stable transfection of *L. tarentolae* was performed as previously described [54]. Briefly, *L. tarentolae* parasites at logarithmic growth phase were suspended in electroporation buffer at a total number of 4×10^7^ cells/400 µl. Pre-cooled parasite suspension mixed with 15 µg of purified linear pLEXSY-PT-GFP (Promega gel extraction kit, USA) in electroporation cuvette, was electroporated (Gene Pulser Xcell, Bio Rad, USA), was recovered in M199 (Sigma, Germany) -10% FCS selection antibiotic free medium and was subsequently plated on Nobel agar plates supplemented with or without 30 µg/ml of G-418 (Gibco, USA). Resistant clones were sub-cultured with 200 µg/ml of G-418 for three consecutive weeks in logarithmic growth phase. Resistant parasites were subject to complementary assays at DNA and RNA levels. Integration of the linear plasmid encoding polytope-EGFP fragment into the chromosome was subsequently confirmed with a set of forward (F3001: TATTCGTTGTCAGATGGCGCAC) and reverse (A1715: GATCTGGTTGATTCTGCCAGTAG) primers (MWG, Germany) each specific for chromosomal and plasmid DNA respectively. Complementary PCRs (polymerase chain reactions) with sets of primers specific for different integrated genes confirmed polytope integration. At the RNA level, after RNA extraction (QIAGEN RNeasy extraction kit-Germany) and following oligo-dT reverse transcriptional cDNA amplification, expression of the polytope was confirmed by EGFP sequence specific primers (EGFP-F: ATGATATCAAGATCTATGGTGAGCAAGGGC, EGFP-R: GCTCTAGATTAGGTACCCTTGTACAGCTCGTC).

### Polytope degradation assay using fluorescent *L. tarentolae*

Resistant parasites under high drug concentration (200 µg/ml) were washed and treated with Proteasome Inhibitor MG132 (Biotrend, Germany) at two different concentrations: 5 µM and 10 µM. Un-treated cells were used as control. After three hours of incubation at 26°C, cells were washed and re-suspended in PBS buffer for microscopic and flow cytometric analysis.

### Mouse Immunization and splenocyte isolation protocol

Endotoxine free recombinant pcDNA-PT was prepared by QIAGEN EndoFree Plasmid Mega Kit, (QIAGEN, Germany) for immunization. Eight weeks-old male HLA-DRB1*0101/HLA-A*0201 transgenic mice were humanly anesthetized and injected with 100 µl diluted Cardiotoxin (Latoxan, France) 4 days before DNA immunization (6.8 µg per mouse). 100 µg Purified plasmid DNA was inoculated bilaterally in hamstring muscles of each anesthetized mice followed by 2 boosters with one week interval each. Ten days after the last booster, mice were humanly euthanized. Dissected spleen from each individual mouse was split into single cell suspension of splenocytes (BD Bioscienses 70 µm cell strainer) and depleted of red blood cells by ACK lyses buffer (Sigma, France). RBC free suspension was washed and re-suspended in x-vivo 15 medium (Lonza, France) for further incubation (3 hours) prior to immunoassays. Both experiments in BALB/c and transgenic mice were repeated twice.

### IFN-γ ELISpot assay

*Ex-vivo* ELISpot was conducted as previously described [55], [56]. Briefly, splenocytes from immunized mice were incubated at 2×10^5^ cells per well in duplicates in ELISpot Anti-IFN-γ coated plates in presence of the relevant or control peptides (Proimmune, Ltd-UK). Plates were incubated for 16 to 18 hours at 37°C, and spots were revealed following the manufacturer's instructions (GenProbe-France). Spot-forming cells (SFC) were counted using C.T.L. Immunospot system (Cellular Technology Ltd. Germany). Stimulations resulting in spots 2 times the negative control (un-stimulated cells) and more than 10 were considered positive. Anti-HLA DR antibody (clone L243, provided by Bernard Maillère laboratory, France) was used to confirm HLA class I restricted response.

### Short term CTL line generation

12×10^6^ splenocytes per well from each individual mouse were plated in 6 well plates (Greiner Bio-one, France) in RPMI-1640 supplemented with 10% fetal bovine serum and 50 µM β-mercaptoethanol. Individual peptides used for *in-vitro* stimulation were supplied at 5 µg/ml final concentration. To amplify responding clones' frequency, IL-2 was added at 100 U/ml final concentration and cells were incubated for three more days. Peptide specific cytotoxic activity of CD8^+^ T cell lines generated during *in-vitro* culture was further assessed in CTL assay.

### Chromium 51(^51^Cr) release assay (CTL assay)

Cytolytic activity was tested in a standard 4-hour ^51^Cr release assay [57]. RMA/s target cells loaded with individual peptides at a 10 µg/ml final concentration and labeled with ^51^Cr radioactive isotope were co-cultured with short term CTL lines making three different effector-to-target ratios. Supernatant fluids from all stimulation conditions were harvested in 96 well Lumaplates (PerkinElmer, USA). Plates were air dried (overnight) and radioactivity was measured in 1450 MicroBeta (Wallac, Finland) the following day. Results are expressed as the mean of triplicates in % of specific lyses: [(experimental − spontaneous release)/(total – spontaneous release)] ×100. An irrelevant HLA-A*0201 restricted 9 mer peptide derived from human telomerase was used as negative control.

### Statistical analysis

Data were analyzed based on variance difference significance. In the case of P-value<0.05, un-paired t-test was used and in the case of P-value>0.05, Mann-Whitney U non-parametric test was used. P-value less than 0.05 was considered significant in each test.

## Results

### Rational design of a polytope construct

**Figure 1:**
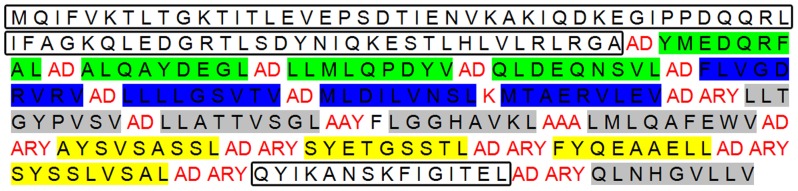
Final arrangement of the polytope construct used for immunization. 13 HLA-A*0201 plus 4 H-2Kd control restricted peptides were arranged in tandem with spacers for accurate proteosomal cleavage (in red). Additional N-terminal ubiquitin sequence (bigger box) for proteosomal degradation, and C-terminal TT_830_ epitope (smaller box) for CD8 T cell response enhancement were also included. Peptides depicted in green are from *Lm*STI-1, peptides in blue from LPG-3, peptides in gray from CPB/CPC and peptides in yellow are H-2Kd restricted (AYS = Kd1, SYE = Kd2, FYQ = Kd3, SYS = Kd4).

**Figure 2:**
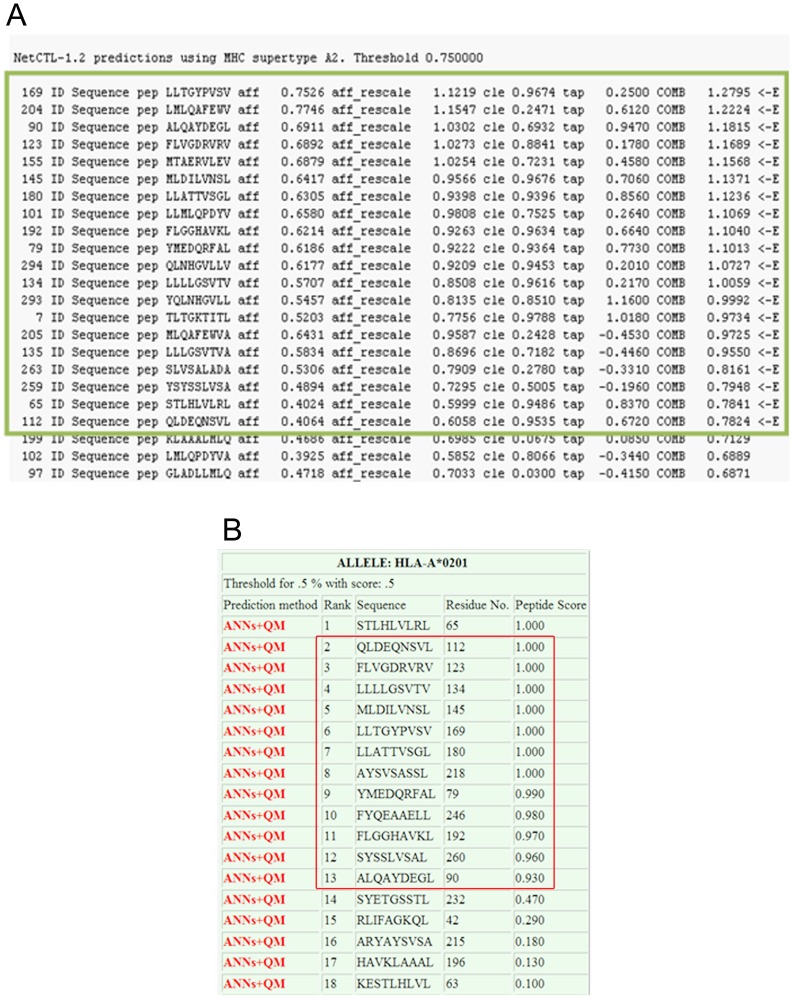
Immunoinformatic prediction of proteosomal cleavage pattern. A. NetCTL cleavage pattern of the final selected polytope sequence. This diagram shows the output frm NetCTL software after proteosomal cleavage with a threshold over 0.75 to discriminate between binders and non-binders. Junctional peptides have negative scores for TAP binding. Peptides scored over threshold are enclosed in a box. B. Immunoproteasome cleavage pattern of the final selected polytope sequence predicted by nHLApred. This diagram shows the output frm nHLApred software after immunoproteosomal cleavage with a threshold over 0.5 to discriminate between binders an non-binders. Peptides scored over threshold are enclosed i a box. First Peptide in the box was an ubiquitin derived peptide.

To design a construct able to stimulate *Leishmania* specific CD8 T cell responses, 13 selected peptides from our previous study plus 4 H-2Kd control restricted peptides were arranged in tandem. Figure 1 depicts the final arrangement of peptides. Besides spacers we had inserted additional extensions as Alanine/Arginine/Tyrosine (ARY) into flanking area of each H-2Kd determinant to increase the peptide affinity for TAP molecule [47], [50], [58]. As illustrated in Figure 2-A (the results of NetCTL analysis) and Figure 2-B (the results of nHLApred analysis), the final arrangement was chopped into desired peptides (top of the list) resulting in least junctional peptides. Since a protein is ubiquitinated only if it carries degradation signals [59], [60] and a polytope is devoid of these signals, the final arrangement was additionally supported with an ubiquitin sequence upstream of the polytope with a G76A substitution to protect deubiquitination by hydrolases. Ubiquitination efficiency was further evaluated by a GFP expressing *Leishmania* species.

Furthermore, TT_830_ sequence, a universal Th1 epitope from tetanus toxoid, was integrated downstream of the polytope sequence to meet the prerequisite of naïve CD8 T cell response activation [61], [62]. Th1 peptide was separated by ARY extension since cytoplasmic Th1 epitope must gain access to secretory cavity to appear in the context of HLA class II molecules [63], [64], [65]. TT_830_ was chosen as helper epitope due to lack of sufficient knowledge on human HLA class II restricted *Leishmania* peptides. Further testing of the polytope construct devoid of TT_830_ will show whether inclusion of such an epitope is necessary.

Hydrophobic/hydrophilic nature of the protein is an additional point which is necessary to be taken into account for efficient expression. Figure S1 depicts the hydrophobicity pattern of the final arrangement analysed by Expasy-Protscale (http://web.expasy.org/protscale/). N-terminal region of the polytope was quite hydrophilic due to ubiquitin sequence. 921 bp long sequence, cloned in pUC57, was subject to a few cloning steps summarized in Figure S2.

### Polytope expression was confirmed in transiently transfected mammalian COS-7 cells

**Figure 3:**
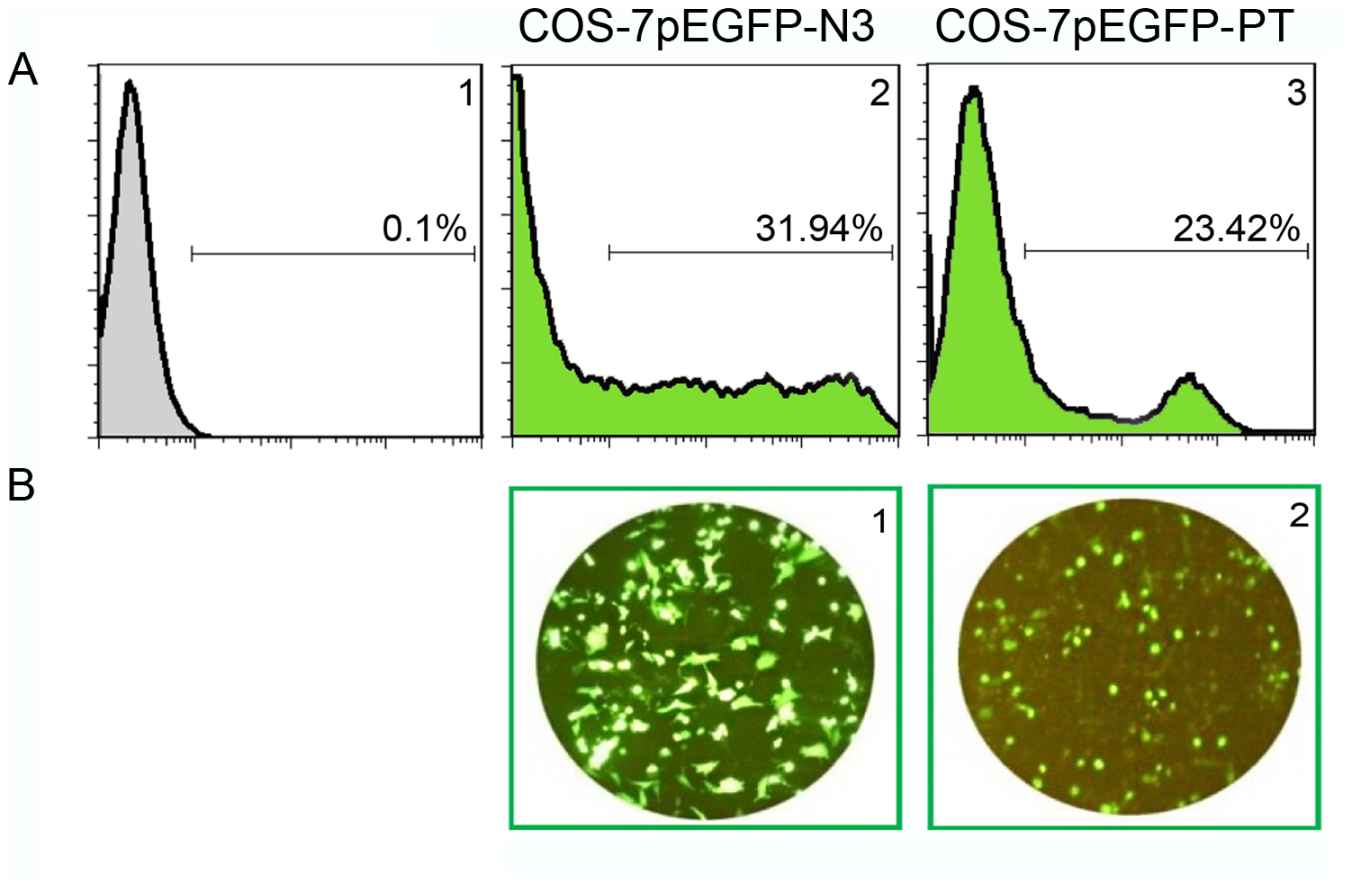
*In-vitro* evaluation of polytope expression using COS-7 cells. Recombinant pEFGP-PT was transiently transfected into COS-7 cells by means of linear Polyethylenimine 25 KDa. A1 represents COS-7 cells without any transfection, A2 represents COS-7 cells transfected with pEGFP-N3 as positive control and A3 represents COS-7 cells transfected with pEGFP-PT. Panel B represents the corresponding microscopic feature of each condition, B1 represents positive control and B2 represents COS-7 cells transfected with pEGFP-PT after 24 hours.

COS-7 cells were transiently transfected with pEGFP-PT to verify the expression of the polytope in eukaryotic cells recognizing CMV promoter. As shown in Figure 3, 24% of pEGFP-PT transfected COS-7 cells were GFP positive. Since the polytope sequence was inserted upstream of the EGFP sequence, fluorescense detection directly correlated with polytope expression. pEGFP-N3 transfected cells with 32% positivity were used as control.

### Proteasome degradation was obviously ubiquitin dependant

**Figure 4:**
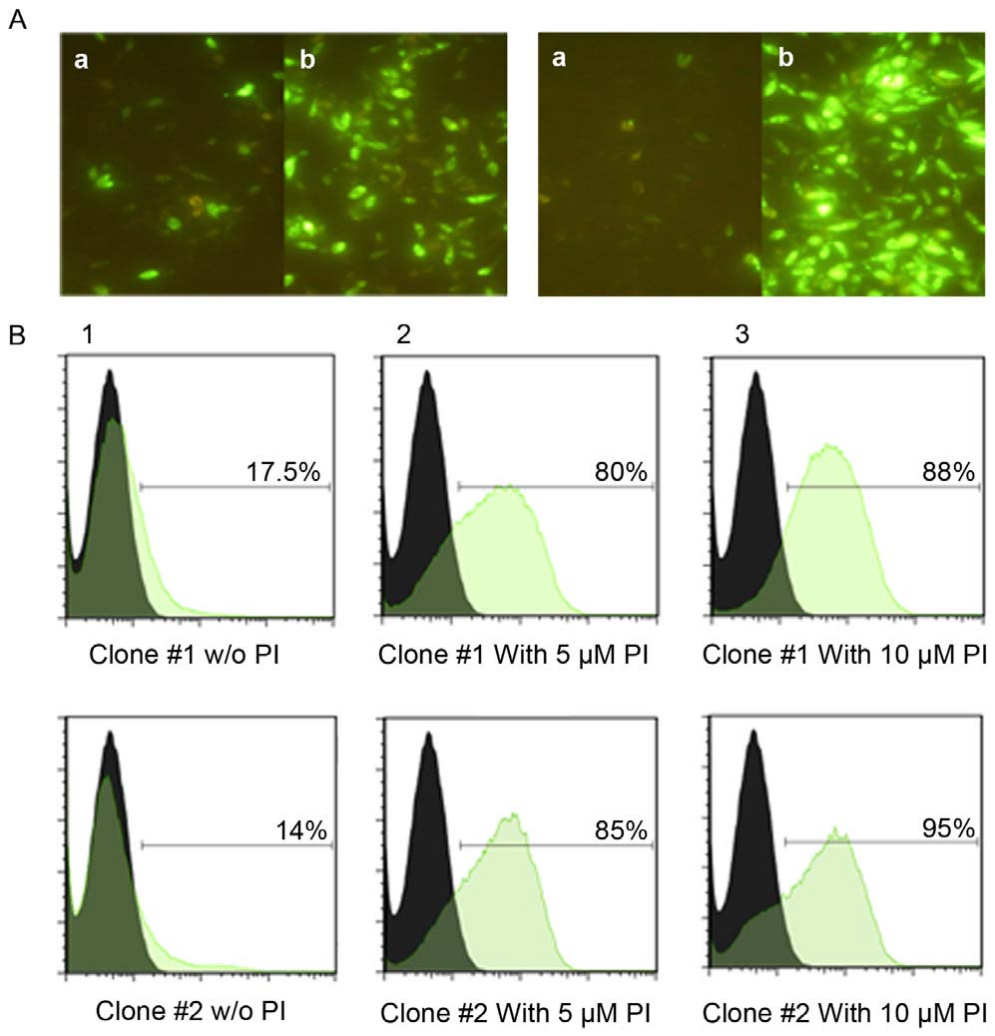
Transfected *Leishmania* parasites before and after treatment with proteasome inhibitor. Stable transfected parasites harboring polytope sequence were generated and used for evaluation of ubiquitinated polytope expression and degradation. A. Shows the fluorescent microscope patterns in 2 microscopic fields. Pale colored parasites before MG132 treatment turned sharp green in the presence of MG132 inhibitor. Part “a” in each field reflects before and part “b” after treatment with 10 µM MG132 for 3 hours. B. Illustrates fluorescent intensity of 2 different transfected clones (Clone#1 and clone #2) before and after treatment with MG132. Column 1: before treatment, column 2: treatment with 5 µM and column 3: treatment with 10 µM of MG132. Numbers on each plot represent the GFP positive population which drastically increases after MG132 treatment. PI: proteasome inhibitor.

In this study we established a GFP-based instead of radioactive degradation detection method [66] to evaluate the efficacy of ubiquitination. In this system, homologous recombination directly inserted the polytope-EGFP (PT-EGFP) sequence with N-terminal ubiquitin into the ribosomal DNA region (r-DNA) of *L. tarentolae* where the high expression levels meet the demands for ribosomal assembly. The plasmid integration and expression was confirmed at both DNA (Figure S3) and RNA level (Figure S4). Stably transfected *L. tarentolae* parasites with pLEXSY-PT-EGFP were cultured three consecutive weeks under G-418 pressure (200 µg/ml) for logarithmic expansion. Recombinant parasites shining green were barely detectable before treatment with a small three-peptide inhibitor which easily disseminates into the cell and transiently disturbs the proteasome function in a competitive manner in contrast to control pLEXSY-EGFP transfected *L. tarentolae* (data not shown). One prominent character of this system was easy handling of parasite in a rather simple medium for weeks in a logarithmic phase to assure a low level of GFP expression. Recombinant clones were then treated with MG132 to transiently stop degradation process. Parasites shining green were easily detectable by flow cytometry and fluorescent microscope monitoring within three hours of treatment (Figure 4). The effect of MG132 treatment on pLEXSY-PT-EGFP transfected clones was compared to another clone transfected with an ubiquitin free construct. The level of expression before and after treatment with proteasome inhibitor roughly differed in latter case compared to ubiquitinated construct (Figure S5). So it was firmly established that ubiquitination successfully directed the polytope to cytoplasmic degradation right after synthesis.

### Polytope expression was confirmed by DNA immunization in BALB/c mice

**Figure 5:**
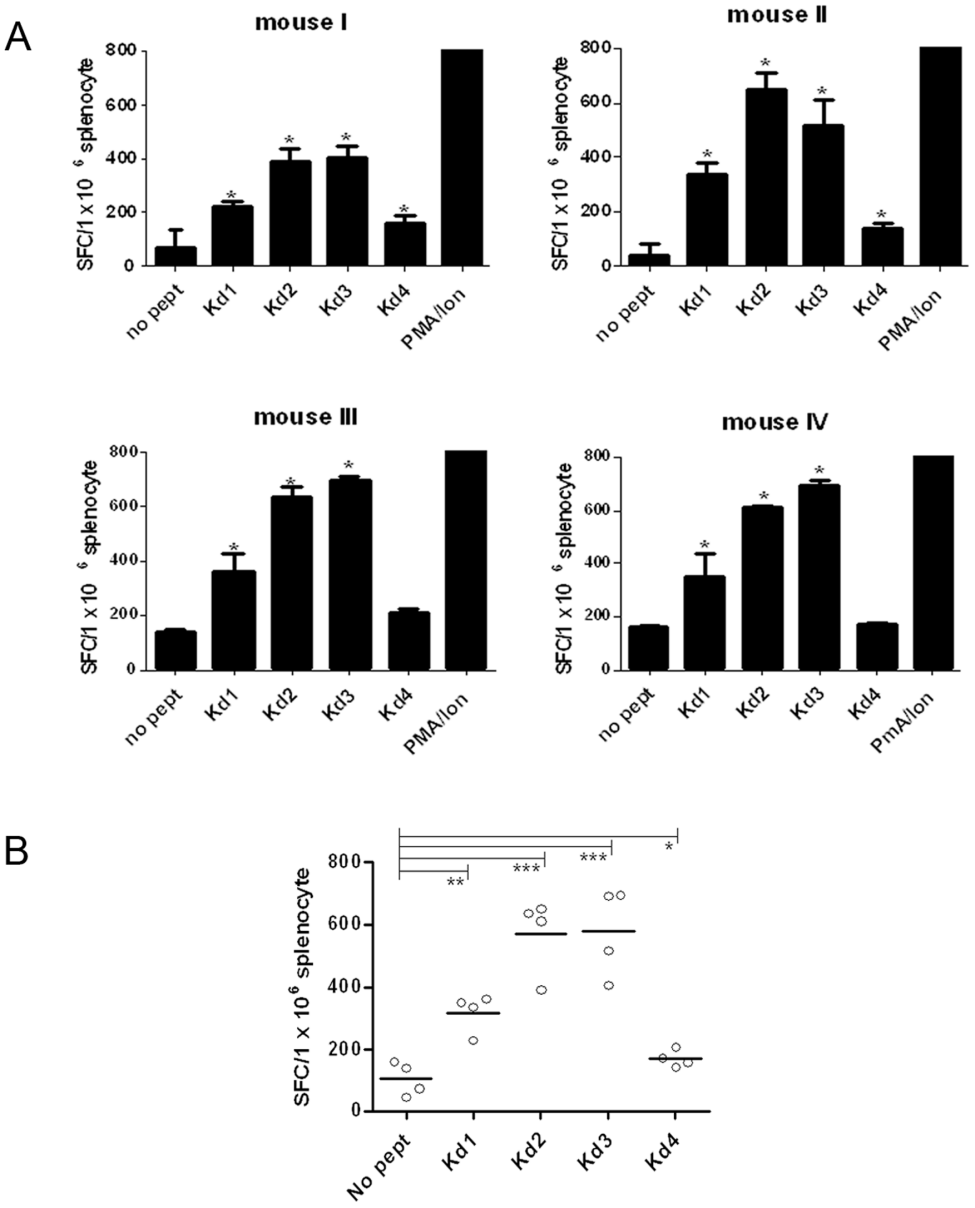
Balb/c response against 4 H-2Kd restricted peptides. 4 mice were immunized with polytope construct three times with one week interval and sacrificed 10 days after the last booster. Splenocytes from individual mice were *in-vitro* re-stimulated by representative peptides (Kd1-4) of Balb/c and specific IFN-γ production was evaluated by *ex-vivo* ELISPOT assay. A. Representative of two experiments. Columns, mean of spots from duplicate wells for each mice from one representative experiment; bars, SD. Stimulations resulting in spots two times the negative control (unstimulated cells) and more than 10 were considered positive (stars). B. Statistical analysis of consolidated data from 4 mice against each individual peptide. The response against all 4 peptides appeared statistically significant compared to un-stimulated control cells (*p*<0.05) with an exception for Kd4 which was subdominant in comparison to the rest. Horizontal lines represent the mean value. SFC: Spot Forming Cells.

Four H-2Kd restricted epitopes (previously introduced by Domunteil *et al.* [67]) were used in a control experiment in BALB/c mice. Figure 5A shows individual IFN**-γ** responses of 4 immunized mice against individual peptides detected by *ex-vivo* ELISpot assay following DNA-DNA prime-boost immunization. The response against all 4 peptides appeared positive and statistically significant (*p*<0.05) compared to un-stimulated control cells. As shown in figure 5B, Kd4 stimulated a significant but weaker IFN**-γ** production compared to the other three examined peptides. These results confirmed that the long polytope was properly expressed, translated and chopped into peptides by proteasome cleavage leading to peptide presentation and priming of naïve CD8^+^ T cells.

### *Leishmania* specific CD8 T cells were induced against HLA-A*0201 restricted peptides in HLA transgenic mice

**Figure 6:**
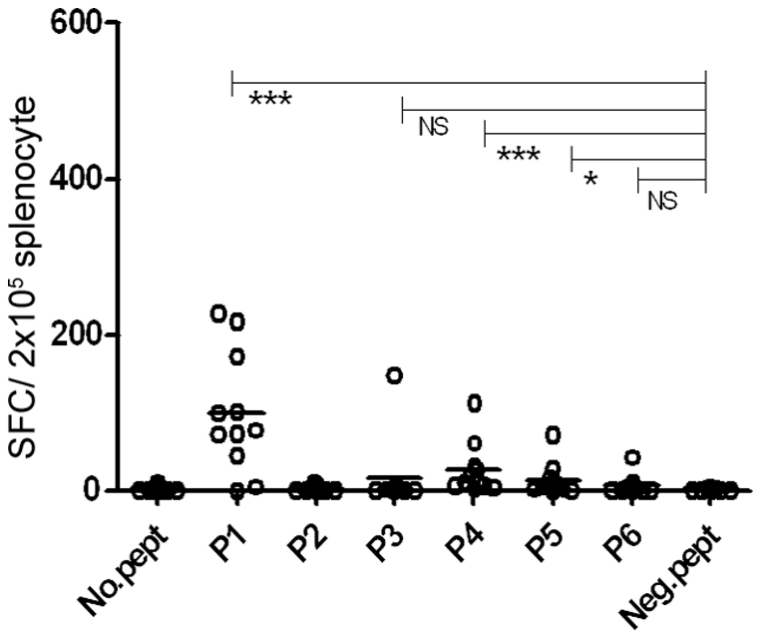
*Ex-vivo* evaluation of the specific response against six peptides (5 µg/ml/peptide) in HLA A2/DR1 mice. A total of 11 mice in two rounds of experiments were immunized with polytope construct three times with one week interval and sacrificed 10 days after the last booster. Splenocytes from individual mice were *in-vitro* re-stimulated by representative peptides (P1–P6) of HLA-A2 and specific IFN-γ production was evaluated by *ex-vivo* ELISPOT assay. Each dot represents mean of duplicate wells for each individual mice response against each peptide. Neg.pept (negative control peptide) represents a 9 mer HLA-A*0201 restricted peptide from human telomerase. Horizontal lines represent the mean value. No.pept =  no peptide stimulation. NS: not-significant.

To evaluate the *in-vivo* immunogenicity of selected peptides, HLA-A2 Transgenic mice were immunized with polytope DNA construct. Immune reactivity was assessed by IFN-γ secretion from peptide stimulated splenocytes. Figure 6 illustrates the results for *ex-vivo* stimulation (16 hrs of culture) of splenocytes from each individual mice against individual HLA-A*0201 restricted peptides at 5 µg/ml/peptide final concentration. IFN**-γ** producing cells were enumerated by ELISpot assay. 10 mice out of 11 responded to 1-3 peptide out of 6 (Figure S6). LPG-3-P1 (P1: LLLLGSVTV) elicited a dominant response compared to *Lm*STI-1-P3 (P3: QLDEQNSVL), *Lm*STI-1-P4 (P4: LLMLQPDYV), LPG-3-P5 (P5: MLDILVNSL) and *Lm*STI-1-P6 (P6: ALQAYDEGL). *Lm*STI-1-P2 (P2: YMEDQRFAL) at this stage provoked no response. The response against P1, P4 and P5 was statistically significant compared to negative control peptide (*p*<0.05).

**Figure 7:**
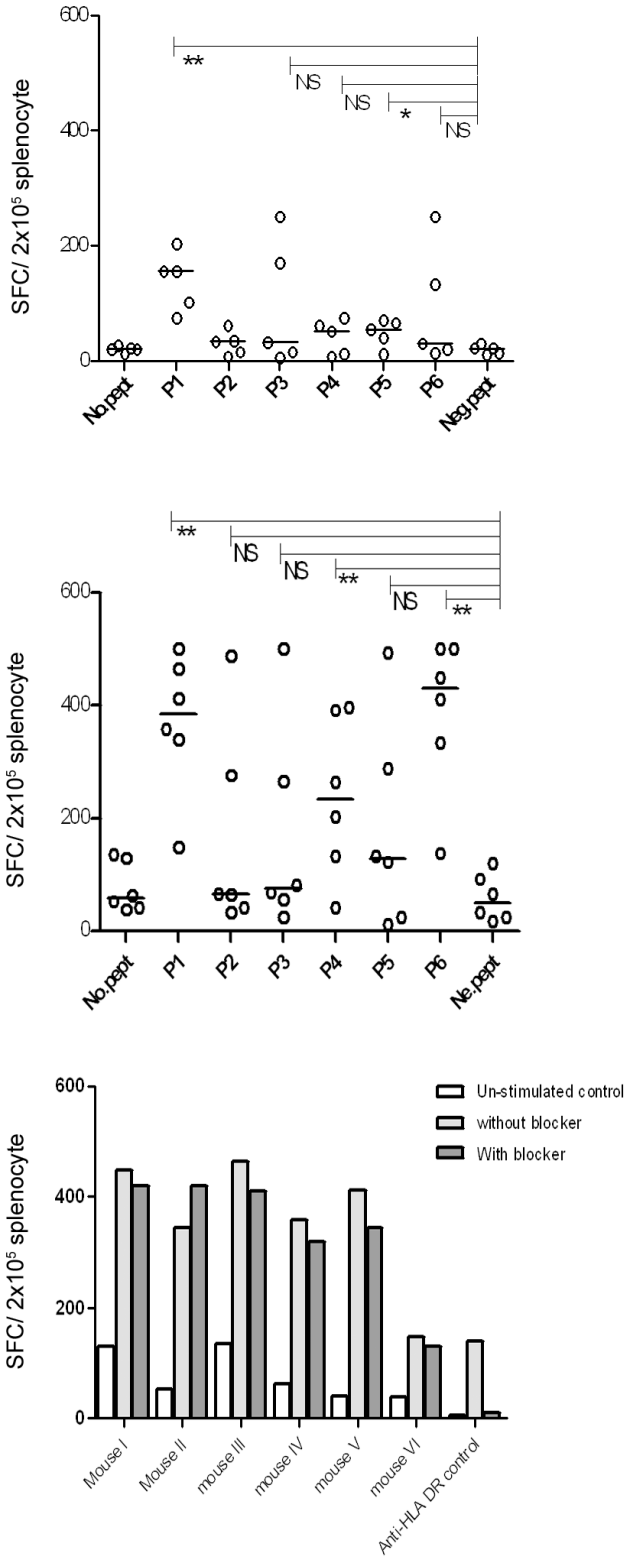
*In vitro* evaluation of the specific response against six peptides in HLA A2/DR1 mice after one week stimulation (with 100 u/ml IL-2). A. Splenocytes from a total of 5 mice immunized with polytope construct three times with one week interval and sacrificed 10 days after the last booster were re-stimulated by representative peptides (5 µg/ml/peptide) of HLA-A2. Specific IFN-γ production was evaluated by *ex-vivo* ELISPOT assay. Each dot represents mean of duplicate wells for each individual mice response against each peptide. B. Splenocytes were stimulated with 10 µg/ml/peptide instead of 5 µg/ml/peptide. Neg.pept (negative control peptide) represents a 9 mer HLA-A*0201 restricted peptide from human telomerase. Horizontal lines represent the mean value. No.pept =  no peptide stimulation. NS: not-significant. C. P1 stimulation of splenocytes from B. along with Anti-HLA-DR (L243) blocker antibody. The response was CD8^+^ T cell restricted since CD4-T cell blockade by anti-HLA-DR, did not influence the outcome. L243 definitely lowers a potent CD4 T cell response in human PBMC against a TERT derived universal MHC class II restricted cancer peptide (UCP).

To further elucidate the immune response under different stimulation conditions, splenocytes were stimulated *in-vitro* with individual peptides (5 µg/ml/peptide) along with IL-2 for one week. Splenocytes from 4 mice out of 5 responded to 2–4 peptides out of 6 (Figure S7). As shown in Figure 7A, an elevated response was detected against peptides P3 and P6. The response against P1 was detected as strong as before, but the response against peptide P2, P4 and P5 was barely affected.

Next the splenocytes were stimulated with a higher concentration of each peptide (10 µg/ml/peptide) during *in-vitro* stimulation. Splenocytes from 5 mice out of 6 responded to 3–6 peptides out of 6 (Figure S8). As shown in Figure 7B an elevated response was detected against all six peptides including P2. The response against P1, P4 and P6 was statistically significant (*p*<0.05) (with 6 mice out of 6 responding to the relevant peptides). The response was CD8^+^ T cell restricted since CD4-T cell blockade by anti-HLA-DR, did not influence the outcome (Figure 7C).

Therefore, we could consider P1 as a dominant high affinity peptide since it elicited potent IFN**-γ** response both *ex-vivo* and *in-vitro* even at low peptide concentration. The remaining tested peptides could be considered as subdominant regarding IFN**-γ** response since the response could be raised by *in-vitro* IL-2 stimulation (P3 and P6) and/or by increasing the level of peptide concentration (P2, P3, P4, P5, P6).

### Specific CD8-T cell lymphocytes displayed cytotoxic activity against peptide loaded target cells

**Figure 8:**
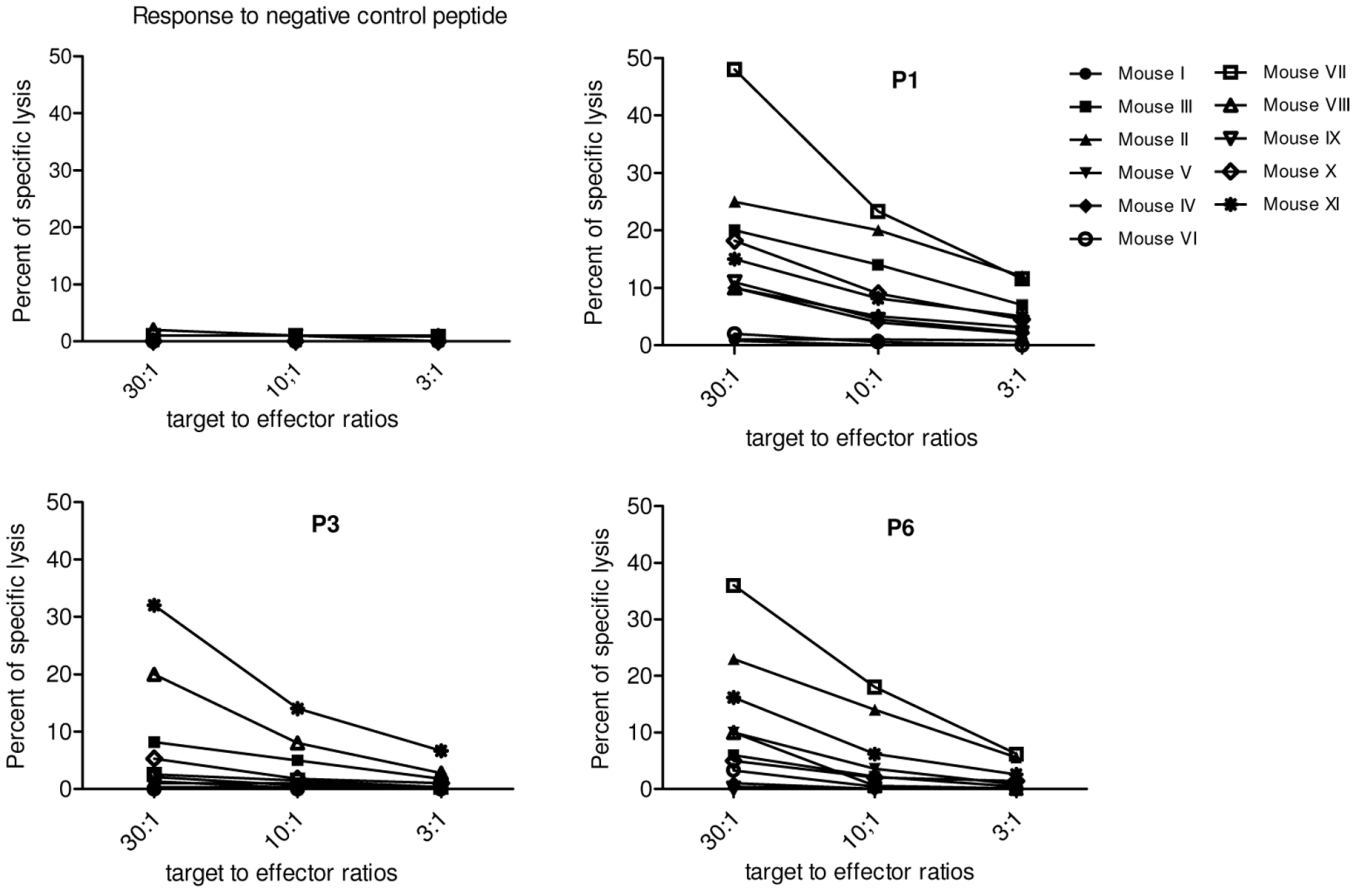
Cytotocic T cell response against RMA/s target cells loaded with individual peptides. Cytolytic activity was tested in a standard 4-hour ^51^Cr release assay. RMA/s target cells loaded with individual peptides at a 10 µg/ml final concentration and labeled with ^51^Cr radioactive isotope were co-cultured with short term CTL lines making three different effector-to-target ratios. Results are expressed as the mean of triplicates in % of specific lyses: [(experimental − spontaneous release)/(total – spontaneous release)] ×100. A 9-mer HLA-A0201 restricted peptide from human telomerase was used as negative control.

The cytolytic activity of the T cell clones against all six peptides was investigated after one week stimulation with 5 µg/ml/peptide and 100 U/ml IL-2. RMA/s cells loaded with relevant peptides served as targets. All the results were compared to a negative control. P1, P3 and P6 induced specific lysis by CTL lines regarding P1 as the most potent inducer. P2, P4 and P5 provoked no cytolysis at all. Figure 8 illustrates the percent of specific lysis of targets loaded by P1, P3 and P6 by T cell clones from individual mice at 3 different effector-to-target (E/T) ratios and Table 2 compares the results at 30∶1 E/T ratio. Thereby 3 out of 6 peptides were considered as Tc1 type cells with both IFN**-γ** production potential and cytolytic activity. This makes the construct quite attractive for vaccination against *Leishmania* infectious challenge.

**Table 2:** Percent of specific lysis of targets loaded by P1, P3 and P6 by T cell clones from individual mice at 30∶1 E/T effector to target ratio.

Protein	Peptide sequence	R/T^a^	Specific lysis (%)^b^ of responders
LPG-3	LLLLGSVTV (P1)	8/11	20, 25, 10, 48, 10, 11, 18, 15
*Lm*STI-1	QLDEQNSVL (P3)	4/11	8.2, 20, 5.3, 32
	ALQAYDEGL (P6)	7/11	6, 23, 10, 36, 10, 5, 16.2

a Responder to tested mice.

b Specific lysis at 30∶1 effector to target ratio.

## Discussion

Today it is believed that CD8^+^ T cells take a significant part in immunity against leishmaniasis. So polytope vaccines might turn a hope in Leishmaniasis control. Therefore in this study we decided to evaluate the immunogenicity of a DNA polytope construct encompassing previously determined immunogenic peptides [43] in a relevant preclinical mouse model. As indicated in our previously published paper, we examined the immune response of CL recovered HLA-A2+ individuals against different peptide pools and included in our DNA construct all peptides which had detectable stimulatory effect on PBMCs (CPB and CPC -5 peptides, *Lm*STI-1 -4 peptides and LPG-3 - 4 peptides). A weaker response to CPB/CPC peptide pool was detected which was implied to be more potentially restricted to other closely related super-types besides A2. In this study we only focused on 6 out of 8 remaining A2 restricted peptides (based on their scores and importance) to check the immunogenicity in HLA-A2 transgenic animals.

A polytope DNA construct was provided sticking to basics of rational design such as inserting spacers between adjacent peptides [68] and targeting the polytope to proteasome enzymatic degradation [69]. We selected DNA immunization instead of peptide immunization (which is deeply dependent on modulators of immunogenicity) or polytope string (which is more immunogenic but less cost effective) because DNA constructs are well appreciated as efficient for stimulating both T-helper-1(Th-1) and T-cytotoxic-1(Tc-1) responses. We first assessed the immunogenicity of the polytope construct in BALB/c mice where 4 out of 4 H-2Kd restricted peptides previously characterized by Dumonteil et al. were shown to be immunogenic [67]. In their experiment, the peptides were directly inoculated into BALB/c mice with adjuvant and ranked in Kd2>Kd3>Kd1>Kd4 order based on ELISA detected IFN**-γ** production. Our results were totally concordant confirming that all 4 peptides were quite accessible for the immune system after proteasome degradation. In this DNA construct the H-2Kd restricted peptides were inserted downstream to the polytope. Therefore it was inferred that the polytope was fully expressed till the end point and was chopped into desired peptides. This result further encouraged us to proceed with HLA-humanized mice which harbor less CD4^+^ and CD8^+^ T cell amounts than conventional mice strains [70].

The moderate efficacy of many vaccine trials primarily reported as protective in wild-type animal models is partly explained by the different influence that human and animal MHC have on the outcome of the immune response [71]. Humanised transgenic mice models expressing human HLA instead of mouse MHC, fill this gap between human and mice. These preclinical models have shown promising results despite subtle differences in antigen processing machinery including proteasome cleavage and TAP molecules affinity for peptides [72], since the immunological hierarchy is approximately the same in both models and about 80% of peptides immunogenic in one are also immunogenic in the other [73].

In our previous study we had focused on peptides in a pool to screen the immunogenicity by human PBMCs. As reported, the response against *Lm*STI-1 peptide pool (4 peptides) appeared in higher frequency and lower potency quite contrary to LPG-3 peptide pool (4 peptides) with higher potency and lower frequency. Here we studied the individual response of 4 peptides in *Lm*STI-1 pool and 2 out of 4 in LPG-3 stimulating pool in a DNA construct. Almost all peptides were able to induce specific CD8 T cell responses *in-vivo*, indicating the adequacy of HLA-A2 transgenic mice T cell repertoire. P1, derived from LPG-3, dominantly induced Tc1 type responses. P3, P4, P6 (derived from *Lm*STI-1) and P5 (the other peptide form LPG-3 group) appeared subdominant. P2 was rather a weak immunogen. In this study P1 from LPG-3 appeared quite dominant regarding IFN-γ production and cytolytic activity compared to peptides from *Lm*STI-1. The level of expression of LPG-3 declines in the amastigote stage of the parasite but *Lm*STI-I is constitutively expressed both in promastigote and amastigote stage of parasite with even higher expression in amastigote stage. So the level of response in human experiment shows a good correlation between affinity, final avidity and immunogenicity of the peptides [55], [74].

We concluded that the hierarchy observed between different peptides is in fact a function of peptide affinity but not the levels of expression or proteasome cleavage. Fig-1 clarifies our declaration since the P1 peptide appears quite at the middle of the sequence so there is no superiority for level of expression. Also as shown in Fig-2A and 2B, the P1 peptide has no superiority to other peptides regarding its position in the list. More importantly all of the 6 peptides examined are separated by “AD” as spacer (Fig-1). Even subtle differences between human and HLA transgenic mice in TAP-peptide affinity (as described by Pascolo *et al*. [72]) is of less importance because HLA-A2 peptide restriction is less TAP dependant than other HLAs. The main positive point for this peptide is that the score predicted by SYFPEITHI immunoinformatics software is higher than the rest. Some studies confine the selection on SYFPEITHI scores over 24 [75], [76] to stringently select dominant peptides restricted to HLA-A*0201. But here we showed that scores over 20 appear quite satisfactory because helped us predict valuable low affinity peptides. Whether a construct composed of both dominant and subdominant epitopes effectively protects against *Leishmania* challenge is open to further investigations [77], [78].

To end with a proper arrangement of 17 peptides (Thirteen HLA-A*0201 restricted peptides and four H-2Kd restricted ones), all possible combinations with or without spacers were examined by immunoinformatic methods. In our experience, arrangements with AAA, AAY and AD spacers had a better performance regarding peptide processing compared to spacer free arrangements and arrangements with lysine (K) as spacer. This is in agreement with the “P1” premise. First experiments with polytope constructs appeared as string of beads without any flanking sequences between each two determinants with detectable immunogenicity for comprising peptides. However in some cases, the immunogenicity was apparently a function of peptide position regarding flanking sequences [79]. The influence of the P1 amino acid (the first residue next to the C-terminus of the peptide) on processing efficiency was elucidated by further investigation. Where some results indicated a preference for natural flanking sequences for proteasome processing [80] others showed that C-terminal flanking of epitopes with alanine increased the epitope processing and recognition by T cells [81], [82].

In our study “AD” was more satisfying as spacer regarding the least junctional peptide criteria in comparison to other two spacers. This might be rather hard to discuss since it is fully dependant on the peptide composition at one side and prediction methods and the algorithms they rely on, on the other side. Since flanking spacers and their nature is rather a controversial subject, immunoinformatic helps compare different options simultaneously saving time and energy but it should be kept in mind that *in-silico* prediction is still at its infancy and needs to grow up with more and more accurate data feeding.

Another important point was C-terminal glycine substitution of ubiquitin molecule (G76) with an alanine moiety to keep off hydrolytic enzymes. Protein-ubiqitin complex is rather unstable and readily disassembles by hydrolytic activity [66]. It has been shown that G76 substitutions simply guarantee ubiquitination [83], [84]. This way the polytope is efficiently targeted to further ubiquitination and final degradation right after synthesis. In our experience G76A substitution worked efficiently to stabilize the complex as shown by the difference before and after MG132 treatment. There are some reports for C-terminal insertion and efficient processing [50], but N-terminal conjugates attract more [85], [86]. This could be simply explained by the fact that ubiquitin molecules bind other proteins and also other ubiquitin residues by their C-terminal glycine.

Here we investigated the efficiency of a polytope DNA construct sticking to the optimal designation criteria in the literature including: N-terminal G76A ubiquitin sequence, proteosomal cleavage considerations and C-terminal universal Tetanus Toxoid T-helper epitope to minimize the cost of study with transgenic animals. But it will be invaluable to further assess the different construct designations with many different options for processing and presentation like N-terminal signal sequence instead of Ubiqitine or *Leishmania* derived HLA-class II restricted peptides instead of TT_830_ and evaluate the level of protection conferred by *Leishmania* challenge in transgenic mice.

In our recent studies we focused on *in-silico* prediction of CD8 stimulating peptides from well known proteins of *Leishmania* and extended the study to experimental *in-vitro* and *in-vivo* evaluations. It was eventually proved that this procedure could result in potential CD8 stimulating peptides identification. To our knowledge, these studies for the first time report HLA class I restricted peptides from *Lm*STI-1 and LPG-3 proteins of *Leishmania*, immunogenic both in human and relevant animal model. Previous works focused on membrane associated proteins such as gp63 [87] and KmP11 [88] and reverse predicted peptides from *Leishmania* genome regarding membrane associated ORFs [89], [90]. These kinds of antigens are postulated to get access to HLA class I system more efficiently. However Dumonteil *et al.* screened out immunogenic H-2Kd peptide epitopes from *L. major* proteome including 8272 annotated proteins without localization considerations [67]. *Lm*STI-1, recently detected in *L. donovani* secretome [91], is an intracellular protein [92] and one of the immunogenic components of Leish-F vaccine. LPG-3 is a highly immunogenic protein [93], [94] which resembles mammalian endoplasmic reticulum chaperone *GRP94* required for phosphoglycan synthesis and localizes to endoplasmic reticulum of *Leishmania* [95]. Reiner *et al*. have recently characterized members of *L. donovani* secretome with a variety of subcellular localizations. In their study nearly one-third of *Leishmania* secreted proteins in exosomes were predicted to be cytoplasmic including ribosomal, nuclear, mitochondrial and glycosomal proteins but not endoplasmic reticulum proteins. Many proteins appear in the secretome without harboring a conventional N-terminal signal sequence as *Lm*STI-1 [96]. Therefore it could be a valuable approach to characterize novel protein antigens with CD8 T cell activating properties, through wide screening of *Leishmania* genome re-focusing on all possible ORFs with or without annotated function and without considering sub-cellular localization.

Our results were promising since at the end we were able to identify at least one dominant high affinity peptide out of 13 previously *in-silico* predicted ones. The rest of the peptides were subdominant with lower affinities but from a very effective vaccine candidate as *Lm*STI-1 and could be manipulated in further studies to altered peptides with even higher affinity. We cannot ignore other potential peptides that could have been missed by *in-silico* predictions or overlooked, but deeply believe that these kinds of studies in high-through-put scales could in fact speed up peptide screening and identification, both CD4 and CD8 stimulating ones, to build up peptide libraries for different *Leishmania* species. This is a prerequisite for further studies in both ways as polytope vaccination and new antigen hunting for *Leishmania* and is hardly achievable by classical peptide selection methods. Obviously we need more accurate prediction tools and more sensitive *in-vitro* detection tests to reach this end. Fortunately HLA transgenic mice are applicable preclinical models helping to speed up immunogenicity analysis in a human related mouse model.

## Supporting Information

Figure S1 **Kate and Dolite analysis (Protscale) of hydrophobic profile of the final polytope arrangement.** This aanlysis was used to finally select between different combinations and shows the pattern of the final selected polytope sequence. No hydrophobic patch was detected specially at the N-terminal region to hinder translation.
(TIF)

Figure S2 **Cloning pathway. 921 bp long polytop (PT) sequence, was codon optimized for optimal expression in mice and received in pUC57 (pUC57-PT).** pEGFP-PT was used to confirm the expression of the sequence in mammalian cells by CMV promoter, pLEXSY-PT-EGFP was used to confirm the stability of the expressed polytope and pcDNA-PT was used for inoculations.
(TIF)

Figure S3 **Representative PCR reactions used to confirm the plasmid integration into the genome from one transfected** ***Leishmania tarentolae*** **clone.** **A**. Schematic representation of genome sequence after plasmid integration into rDNA *ssu* region. **B**. Full set of PCR reactions followed to confirm the integration at DNA level. Lane 1 and 6: 1 kb DNA ladder marker, lane 2: EGFP fragment (727 bp), lane 3: Polytope fragment (929 bp), lane 4: Polytope-EGFP fragment (1665 bp), lane 7: *SSU* fragment (1070 bp) and lane 8: EGFP-SSU (3000 bp). Lane 7 points to the most important reaction with 2 primers specific for a chromosomal sequence and plasmid sequence with *ssu* fragment in between. Lane 5 refers to un-transfected cells confirmed with F3001/A1715 PCR reaction.
(TIF)

Figure S4 **RNA expression evaluation with a set of primers specific for EGFP.** Lane 1: Fermentas 1 Kb ladder marker, lane 2 and 3: RT-PCR reaction from 2 transfected clones, lane 3: un-transfected cells.
(TIF)

Figure S5 **Effect of MG132 treatment on ubiquitinated and non-ubiquitinated constructs.** **A**. un-transfected parasite, **B**. EGFP transfected parasite, **C** and **D**, *L. tarentolae* transfected with ubiqitinated construct (pLEXSY-PT-EGFP). **E** and **F**, *L. tarentolae* transfected with un-ubiquitinated construct (pLEXSY-A2-EGFP). Expression level of EGFP before and after treatment with proteasome inhibitor roughly differs for un-ubiqitinated protein quite contrary to ubiquitinated protein. Numbers on each plot represent GFP positive population PI: proteasome inhibitor.
(TIF)

Figure S6 ***Ex-vivo*** **response of individual mice against six peptides (5 µg/ml/peptide) in HLA A2/DR1 mice.** A total of 11 mice in two rounds of experiments were immunized with polytope construct three times with one week interval and sacrificed 10 days after the last booster. Splenocytes from individual mice were *in-vitro* re-stimulated by representative peptides (P1-P6) of HLA-A2 and specific IFN-γ production was evaluated by *ex-vivo* ELISPOT assay. Each column represents the mean of duplicate wells stimulated with each peptide. Numbers on each plot show the number of peptides with positive response for each mouse. Peptide stimulations resulting in spots two times the negative control (Neg.pept) and more than 10 were considered positive (stars). Neg.pept (negative control peptide) represents a 9mer HLA-A*0201 restricted peptide from human telomerase.
(TIF)

Figure S7 ***In vitro*** **evaluation of the specific response against six peptides in HLA A2/DR1 mice after one week stimulation (with 100 u/ml IL-2).** Splenocytes from a total of 5 mice immunized with polytope construct three times with one week interval and sacrificed 10 days after the last booster were re-stimulated by representative peptides (5 µg/ml/peptide) of HLA-A2. Specific IFN-γ production was evaluated by *ex-vivo* ELISPOT assay. Each column represents mean of duplicate wells for each individual mice response against each peptide. Numbers on each plot show the number of peptides with positive response for each mouse. Peptide stimulations resulting in spots two times the negative control (Neg.pept) and more than 10 were considered positive (stars).
(TIF)

Figure S8 ***In vitro*** **evaluation of the specific response against six peptides in HLA A2/DR1 mice after one week stimulation with 100 u/ml IL-2 and higher concentration of peptides.** Splenocytes were stimulated with 10 µg/ml/peptide instead of 5 µg/ml/peptide. Numbers on each plot show the number of peptides with positive response for each mouse. Peptide stimulations resulting in spots two times the negative control (Neg.pept) and more than 10 were considered positive (stars).
(TIF)

## References

[1] Organization WH (2010) Control of the leishmaniases. World Health Organization technical report series: xii 21485694

[2] Alvar J, Velez ID, Bern C, Herrero M, Desjeux P, et al (2012) Leishmaniasis worldwide and global estimates of its incidence. PLoS One 7: e35671. 22693548 10.1371/journal.pone.0035671 PMC3365071

[3] Croft SL, Olliaro P (2011) Leishmaniasis chemotherapy–challenges and opportunities. Clin Microbiol Infect 17: 1478–1483. 21933306 10.1111/j.1469-0691.2011.03630.x

[4] Kishore K, Kumar V, Kesari S, Dinesh DS, Kumar AJ, et al (2006) Vector control in leishmaniasis. Indian J Med Res 123: 467–472. 16778324

[5] Kedzierski L (2010) Leishmaniasis Vaccine: Where are We Today? J Glob Infect Dis 2: 177–185. 20606974 10.4103/0974-777X.62881 PMC2889658

[6] Modabber F (2010) Leishmaniasis vaccines: past, present and future. Int J Antimicrob Agents 36 Suppl 1S58–61. 10.1016/j.ijantimicag.2010.06.024 20801000

[7] Okwor I, Mou Z, Liu D, Uzonna J (2012) Protective immunity and vaccination against cutaneous leishmaniasis. Front Immunol 3: 128. 22661975 10.3389/fimmu.2012.00128 PMC3361738

[8] Beaumier CM, Gillespie PM, Hotez PJ, Bottazzi ME (2013) New vaccines for neglected parasitic diseases and dengue. Translational Research 162: 144–155. 23578479 10.1016/j.trsl.2013.03.006

[9] Velez ID, Gilchrist K, Martinez S, Ramirez-Pineda JR, Ashman JA, et al (2009) Safety and immunogenicity of a defined vaccine for the prevention of cutaneous leishmaniasis. Vaccine 28: 329–337. 19879995 10.1016/j.vaccine.2009.10.045

[10] Nascimento E, Fernandes DF, Vieira EP, Campos-Neto A, Ashman JA, et al (2010) A clinical trial to evaluate the safety and immunogenicity of the LEISH-F1+ MPL-SE vaccine when used in combination with meglumine antimoniate for the treatment of cutaneous leishmaniasis. Vaccine 28: 6581–6587. 20688040 10.1016/j.vaccine.2010.07.063

[11] Mougneau E, Bihl F, Glaichenhaus N (2011) Cell biology and immunology of Leishmania. Immunol Rev 240: 286–296. 21349100 10.1111/j.1600-065X.2010.00983.x

[12] Nylen S, Gautam S (2010) Immunological perspectives of leishmaniasis. J Glob Infect Dis 2: 135–146. 20606969 10.4103/0974-777X.62876 PMC2889653

[13] Herath S, Kropf P, Muller I (2003) Cross-talk between CD8(+) and CD4(+) T cells in experimental cutaneous leishmaniasis: CD8(+) T cells are required for optimal IFN-gamma production by CD4(+) T cells. Parasite Immunol 25: 559–567. 15053777 10.1111/j.0141-9838.2004.00668.x

[14] Ruiz JH, Becker I (2007) CD8 cytotoxic T cells in cutaneous leishmaniasis. Parasite Immunol 29: 671–678. 18042173 10.1111/j.1365-3024.2007.00991.x

[15] Muller I (1992) Role of T cell subsets during the recall of immunologic memory to Leishmania major. Eur J Immunol 22: 3063–3069. 1359969 10.1002/eji.1830221206

[16] Muller I, Kropf P, Etges RJ, Louis JA (1993) Gamma interferon response in secondary Leishmania major infection: role of CD8+ T cells. Infect Immun 61: 3730–3738. 8359894 10.1128/iai.61.9.3730-3738.1993 PMC281071

[17] Muller I, Kropf P, Louis JA, Milon G (1994) Expansion of gamma interferon-producing CD8+ T cells following secondary infection of mice immune to Leishmania major. Infect Immun 62: 2575–2581. 8188380 10.1128/iai.62.6.2575-2581.1994 PMC186547

[18] Huber M, Timms E, Mak TW, Rollinghoff M, Lohoff M (1998) Effective and long-lasting immunity against the parasite Leishmania major in CD8-deficient mice. Infect Immun 66: 3968–3970. 9673288 10.1128/iai.66.8.3968-3970.1998 PMC108466

[19] Overath P, Harbecke D (1993) Course of Leishmania infection in beta 2-microglobulin-deficient mice. Immunol Lett 37: 13–17. 7901152 10.1016/0165-2478(93)90126-m

[20] Wang ZE, Reiner SL, Hatam F, Heinzel FP, Bouvier J, et al (1993) Targeted activation of CD8 cells and infection of beta 2-microglobulin-deficient mice fail to confirm a primary protective role for CD8 cells in experimental leishmaniasis. J Immunol 151: 2077–2086. 8102158

[21] Belkaid Y, Von Stebut E, Mendez S, Lira R, Caler E, et al (2002) CD8+ T cells are required for primary immunity in C57BL/6 mice following low-dose, intradermal challenge with Leishmania major. J Immunol 168: 3992–4000. 11937556 10.4049/jimmunol.168.8.3992

[22] Uzonna JE, Joyce KL, Scott P (2004) Low dose Leishmania major promotes a transient T helper cell type 2 response that is down-regulated by interferon gamma-producing CD8+ T cells. J Exp Med 199: 1559–1566. 15184505 10.1084/jem.20040172 PMC2211781

[23] Nateghi Rostami M, Keshavarz H, Edalat R, Sarrafnejad A, Shahrestani T, et al (2010) CD8+ T cells as a source of IFN-gamma production in human cutaneous leishmaniasis. PLoS Negl Trop Dis 4: e845. 20967288 10.1371/journal.pntd.0000845 PMC2953482

[24] Barral-Netto M, Barral A, Brodskyn C, Carvalho EM, Reed SG (1995) Cytotoxicity in human mucosal and cutaneous leishmaniasis. Parasite Immunol 17: 21–28. 7731732 10.1111/j.1365-3024.1995.tb00962.x

[25] Faria DR, Souza PE, Duraes FV, Carvalho EM, Gollob KJ, et al (2009) Recruitment of CD8(+) T cells expressing granzyme A is associated with lesion progression in human cutaneous leishmaniasis. Parasite Immunol 31: 432–439. 19646207 10.1111/j.1365-3024.2009.01125.x PMC2764276

[26] Machado P, Kanitakis J, Almeida R, Chalon A, Araujo C, et al (2002) Evidence of in situ cytotoxicity in American cutaneous leishmaniasis. Eur J Dermatol 12: 449–451. 12370132

[27] Rogers KA, Titus RG (2004) Characterization of the early cellular immune response to Leishmania major using peripheral blood mononuclear cells from Leishmania-naive humans. Am J Trop Med Hyg 71: 568–576. 15569786

[28] Russo DM, Chakrabarti P, Higgins AY (1999) Leishmania: naive human T cells sensitized with promastigote antigen and IL-12 develop into potent Th1 and CD8(+) cytotoxic effectors. Exp Parasitol 93: 161–170. 10529358 10.1006/expr.1999.4452

[29] Hernandez-Ruiz J, Salaiza-Suazo N, Carrada G, Escoto S, Ruiz-Remigio A, et al (2010) CD8 cells of patients with diffuse cutaneous leishmaniasis display functional exhaustion: the latter is reversed, in vitro, by TLR2 agonists. PLoS Negl Trop Dis 4: e871. 21072232 10.1371/journal.pntd.0000871 PMC2970528

[30] Joshi T, Rodriguez S, Perovic V, Cockburn IA, Stager S (2009) B7-H1 blockade increases survival of dysfunctional CD8(+) T cells and confers protection against Leishmania donovani infections. PLoS Pathog 5: e1000431. 19436710 10.1371/journal.ppat.1000431 PMC2674929

[31] Faria DR, Gollob KJ, Barbosa J Jr, Schriefer A, Machado PR, et al (2005) Decreased in situ expression of interleukin-10 receptor is correlated with the exacerbated inflammatory and cytotoxic responses observed in mucosal leishmaniasis. Infect Immun 73: 7853–7859. 16299275 10.1128/IAI.73.12.7853-7859.2005 PMC1307048

[32] Gaze ST, Dutra WO, Lessa M, Lessa H, Guimaraes LH, et al (2006) Mucosal leishmaniasis patients display an activated inflammatory T-cell phenotype associated with a nonbalanced monocyte population. Scand J Immunol 63: 70–78. 16398703 10.1111/j.1365-3083.2005.01707.x

[33] Cong H, Mui EJ, Witola WH, Sidney J, Alexander J, et al (2010) Human immunome, bioinformatic analyses using HLA supermotifs and the parasite genome, binding assays, studies of human T cell responses, and immunization of HLA-A*1101 transgenic mice including novel adjuvants provide a foundation for HLA-A03 restricted CD8+T cell epitope based, adjuvanted vaccine protective against Toxoplasma gondii. Immunome Res 6: 12. 21129215 10.1186/1745-7580-6-12 PMC3009956

[34] Geluk A, van den Eeden SJ, Dijkman K, Wilson L, Kim HJ, et al (2011) ML1419c peptide immunization induces Mycobacterium leprae-specific HLA-A*0201-restricted CTL in vivo with potential to kill live mycobacteria. J Immunol 187: 1393–1402. 21705623 10.4049/jimmunol.1100980 PMC3140574

[35] Mudd PA, Martins MA, Ericsen AJ, Tully DC, Power KA, et al (2012) Vaccine-induced CD8+ T cells control AIDS virus replication. Nature 491: 129–133. 23023123 10.1038/nature11443 PMC3883109

[36] Brinkman JA, Fausch SC, Weber JS, Kast WM (2004) Peptide-based vaccines for cancer immunotherapy. Expert Opin Biol Ther 4: 181–198. 14998777 10.1517/14712598.4.2.181

[37] Perez SA, von Hofe E, Kallinteris NL, Gritzapis AD, Peoples GE, et al (2010) A new era in anticancer peptide vaccines. Cancer 116: 2071–2080. 20187092 10.1002/cncr.24988

[38] Colmenares M, Kima PE, Samoff E, Soong L, McMahon-Pratt D (2003) Perforin and gamma interferon are critical CD8+ T-cell-mediated responses in vaccine-induced immunity against Leishmania amazonensis infection. Infect Immun 71: 3172–3182. 12761096 10.1128/IAI.71.6.3172-3182.2003 PMC155724

[39] Gurunathan S, Stobie L, Prussin C, Sacks DL, Glaichenhaus N, et al (2000) Requirements for the maintenance of Th1 immunity in vivo following DNA vaccination: a potential immunoregulatory role for CD8+ T cells. J Immunol 165: 915–924. 10878366 10.4049/jimmunol.165.2.915

[40] Mendez S, Belkaid Y, Seder RA, Sacks D (2002) Optimization of DNA vaccination against cutaneous leishmaniasis. Vaccine 20: 3702–3708. 12399198 10.1016/s0264-410x(02)00376-6

[41] Mendez S, Gurunathan S, Kamhawi S, Belkaid Y, Moga MA, et al (2001) The potency and durability of DNA- and protein-based vaccines against Leishmania major evaluated using low-dose, intradermal challenge. J Immunol 166: 5122–5128. 11290794 10.4049/jimmunol.166.8.5122

[42] Stager S, Rafati S (2012) CD8(+) T cells in leishmania infections: friends or foes? Front Immunol 3: 5. 22566891 10.3389/fimmu.2012.00005 PMC3342007

[43] Seyed N, Zahedifard F, Safaiyan S, Gholami E, Doustdari F, et al (2011) In silico analysis of six known Leishmania major antigens and in vitro evaluation of specific epitopes eliciting HLA-A2 restricted CD8 T cell response. PLoS Negl Trop Dis 5: e1295. 21909442 10.1371/journal.pntd.0001295 PMC3167772

[44] Oseroff C, Sette A, Wentworth P, Celis E, Maewal A, et al (1998) Pools of lipidated HTL-CTL constructs prime for multiple HBV and HCV CTL epitope responses. Vaccine 16: 823–833. 9627940 10.1016/s0264-410x(97)00264-8

[45] Toes RE, Hoeben RC, van der Voort EI, Ressing ME, van der Eb AJ, et al (1997) Protective anti-tumor immunity induced by vaccination with recombinant adenoviruses encoding multiple tumor-associated cytotoxic T lymphocyte epitopes in a string-of-beads fashion. Proc Natl Acad Sci U S A 94: 14660–14665. 9405669 10.1073/pnas.94.26.14660 PMC25085

[46] Velders MP, Weijzen S, Eiben GL, Elmishad AG, Kloetzel PM, et al (2001) Defined flanking spacers and enhanced proteolysis is essential for eradication of established tumors by an epitope string DNA vaccine. J Immunol 166: 5366–5373. 11313372 10.4049/jimmunol.166.9.5366

[47] Huebener N, Fest S, Strandsby A, Michalsky E, Preissner R, et al (2008) A rationally designed tyrosine hydroxylase DNA vaccine induces specific antineuroblastoma immunity. Mol Cancer Ther 7: 2241–2251. 18645033 10.1158/1535-7163.MCT-08-0109

[48] Li X, Yang X, Jiang Y, Liu J (2005) A novel HBV DNA vaccine based on T cell epitopes and its potential therapeutic effect in HBV transgenic mice. Int Immunol 17: 1293–1302. 16113237 10.1093/intimm/dxh305

[49] Pinchuk I, Starcher BC, Livingston B, Tvninnereim A, Wu S, et al (2005) A CD8+ T cell heptaepitope minigene vaccine induces protective immunity against Chlamydia pneumoniae. J Immunol 174: 5729–5739. 15843575 10.4049/jimmunol.174.9.5729

[50] Bazhan SI, Karpenko LI, Ilyicheva TN, Belavin PA, Seregin SV, et al (2010) Rational design based synthetic polyepitope DNA vaccine for eliciting HIV-specific CD8+ T cell responses. Mol Immunol 47: 1507–1515. 20189249 10.1016/j.molimm.2010.01.020

[51] Larsen MV, Lundegaard C, Lamberth K, Buus S, Brunak S, et al (2005) An integrative approach to CTL epitope prediction: a combined algorithm integrating MHC class I binding, TAP transport efficiency, and proteasomal cleavage predictions. Eur J Immunol 35: 2295–2303. 15997466 10.1002/eji.200425811

[52] Bhasin M, Raghava GP (2007) A hybrid approach for predicting promiscuous MHC class I restricted T cell epitopes. J Biosci 32: 31–42. 17426378 10.1007/s12038-007-0004-5

[53] Doroud D, Zahedifard F, Vatanara A, Najafabadi AR, Taslimi Y, et al (2011) Delivery of a cocktail DNA vaccine encoding cysteine proteinases type I, II and III with solid lipid nanoparticles potentiate protective immunity against Leishmania major infection. J Control Release 153: 154–162. 21530597 10.1016/j.jconrel.2011.04.011

[54] Bolhassani A, Taheri T, Taslimi Y, Zamanilui S, Zahedifard F, et al (2011) Fluorescent Leishmania species: development of stable GFP expression and its application for in vitro and in vivo studies. Exp Parasitol 127: 637–645. 21187086 10.1016/j.exppara.2010.12.006

[55] Adotevi O, Mollier K, Neuveut C, Cardinaud S, Boulanger E, et al (2006) Immunogenic HLA-B*0702-restricted epitopes derived from human telomerase reverse transcriptase that elicit antitumor cytotoxic T-cell responses. Clin Cancer Res 12: 3158–3167. 16707616 10.1158/1078-0432.CCR-05-2647

[56] Adotevi O, Mollier K, Neuveut C, Dosset M, Ravel P, et al (2010) Targeting human telomerase reverse transcriptase with recombinant lentivector is highly effective to stimulate antitumor CD8 T-cell immunity in vivo. Blood 115: 3025–3032. 20130242 10.1182/blood-2009-11-253641

[57] Rohrlich PS, Cardinaud S, Firat H, Lamari M, Briand P, et al (2003) HLA-B*0702 transgenic, H-2KbDb double-knockout mice: phenotypical and functional characterization in response to influenza virus. Int Immunol 15: 765–772. 12750360 10.1093/intimm/dxg073

[58] Burgevin A, Saveanu L, Kim Y, Barilleau E, Kotturi M, et al (2008) A detailed analysis of the murine TAP transporter substrate specificity. PLoS One 3: e2402. 18545702 10.1371/journal.pone.0002402 PMC2408963

[59] Mogk A, Schmidt R, Bukau B (2007) The N-end rule pathway for regulated proteolysis: prokaryotic and eukaryotic strategies. Trends Cell Biol 17: 165–172. 17306546 10.1016/j.tcb.2007.02.001

[60] Sewell DA, Shahabi V, Gunn GR 3rd, Pan ZK, Dominiecki ME, et al (2004) Recombinant Listeria vaccines containing PEST sequences are potent immune adjuvants for the tumor-associated antigen human papillomavirus-16 E7. Cancer Res 64: 8821–8825. 15604239 10.1158/0008-5472.CAN-04-1958

[61] Cho HI, Celis E (2012) Design of immunogenic and effective multi-epitope DNA vaccines for melanoma. Cancer Immunol Immunother 61: 343–351. 21915800 10.1007/s00262-011-1110-7 PMC4019994

[62] Iurescia S, Fioretti D, Fazio VM, Rinaldi M (2012) Epitope-driven DNA vaccine design employing immunoinformatics against B-cell lymphoma: a biotech's challenge. Biotechnol Adv 30: 372–383. 21745560 10.1016/j.biotechadv.2011.06.020

[63] Dani A, Chaudhry A, Mukherjee P, Rajagopal D, Bhatia S, et al (2004) The pathway for MHCII-mediated presentation of endogenous proteins involves peptide transport to the endo-lysosomal compartment. J Cell Sci 117: 4219–4230. 15316082 10.1242/jcs.01288

[64] Lich JD, Elliott JF, Blum JS (2000) Cytoplasmic processing is a prerequisite for presentation of an endogenous antigen by major histocompatibility complex class II proteins. J Exp Med 191: 1513–1524. 10790426 10.1084/jem.191.9.1513 PMC2213437

[65] Tewari MK, Sinnathamby G, Rajagopal D, Eisenlohr LC (2005) A cytosolic pathway for MHC class II-restricted antigen processing that is proteasome and TAP dependent. Nat Immunol 6: 287–294. 15711549 10.1038/ni1171

[66] Rodriguez F, Zhang J, Whitton JL (1997) DNA immunization: ubiquitination of a viral protein enhances cytotoxic T-lymphocyte induction and antiviral protection but abrogates antibody induction. J Virol 71: 8497–8503. 9343207 10.1128/jvi.71.11.8497-8503.1997 PMC192313

[67] Herrera-Najera C, Pina-Aguilar R, Xacur-Garcia F, Ramirez-Sierra MJ, Dumonteil E (2009) Mining the Leishmania genome for novel antigens and vaccine candidates. Proteomics 9: 1293–1301. 19206109 10.1002/pmic.200800533

[68] Babe LM, Chen Y, Chesnut R, DeYoung LM, Huang MT, et al (2009) Optimized multi-epitope constructs and uses thereof. Google Patents

[69] Fu F, Li X, Lang Y, Yang Y, Tong G, et al (2011) Co-expression of ubiquitin gene and capsid protein gene enhances the potency of DNA immunization of PCV2 in mice. Virol J 8: 264. 21624113 10.1186/1743-422X-8-264 PMC3135555

[70] Pajot A, Schnuriger A, Moris A, Rodallec A, Ojcius DM, et al (2007) The Th1 immune response against HIV-1 Gag p24-derived peptides in mice expressing HLA-A02.01 and HLA-DR1. Eur J Immunol 37: 2635–2644. 17668896 10.1002/eji.200636819

[71] Pajot A, Michel ML, Fazilleau N, Pancre V, Auriault C, et al (2004) A mouse model of human adaptive immune functions: HLA-A2.1-/HLA-DR1-transgenic H-2 class I-/class II-knockout mice. Eur J Immunol 34: 3060–3069. 15468058 10.1002/eji.200425463

[72] Pascolo S (2005) HLA class I transgenic mice: development, utilisation and improvement. Expert Opin Biol Ther 5: 919–938. 16018738 10.1517/14712598.5.7.919

[73] Firat H, Garcia-Pons F, Tourdot S, Pascolo S, Scardino A, et al (1999) H-2 class I knockout, HLA-A2.1-transgenic mice: a versatile animal model for preclinical evaluation of antitumor immunotherapeutic strategies. Eur J Immunol 29: 3112–3121. 10540322 10.1002/(SICI)1521-4141(199910)29:10<3112::AID-IMMU3112>3.0.CO;2-Q

[74] Sette A, Vitiello A, Reherman B, Fowler P, Nayersina R, et al (1994) The relationship between class I binding affinity and immunogenicity of potential cytotoxic T cell epitopes. J Immunol 153: 5586–5592. 7527444

[75] Gomez-Nunez M, Pinilla-Ibarz J, Dao T, May RJ, Pao M, et al (2006) Peptide binding motif predictive algorithms correspond with experimental binding of leukemia vaccine candidate peptides to HLA-A*0201 molecules. Leuk Res 30: 1293–1298. 16533527 10.1016/j.leukres.2006.02.010

[76] Elkington R, Walker S, Crough T, Menzies M, Tellam J, et al (2003) Ex vivo profiling of CD8+-T-cell responses to human cytomegalovirus reveals broad and multispecific reactivities in healthy virus carriers. J Virol 77: 5226–5240. 12692225 10.1128/JVI.77.9.5226-5240.2003 PMC153951

[77] Dominguez MR, Silveira EL, de Vasconcelos JR, de Alencar BC, Machado AV, et al (2011) Subdominant/cryptic CD8 T cell epitopes contribute to resistance against experimental infection with a human protozoan parasite. PLoS One 6: e22011. 21779365 10.1371/journal.pone.0022011 PMC3136500

[78] Im EJ, Hong JP, Roshorm Y, Bridgeman A, Letourneau S, et al (2011) Protective efficacy of serially up-ranked subdominant CD8+ T cell epitopes against virus challenges. PLoS Pathog 7: e1002041. 21625575 10.1371/journal.ppat.1002041 PMC3098219

[79] Livingston BD, Newman M, Crimi C, McKinney D, Chesnut R, et al (2001) Optimization of epitope processing enhances immunogenicity of multiepitope DNA vaccines. Vaccine 19: 4652–4660. 11535313 10.1016/s0264-410x(01)00233-x

[80] Neisig A, Roelse J, Sijts AJ, Ossendorp F, Feltkamp MC, et al (1995) Major differences in transporter associated with antigen presentation (TAP)-dependent translocation of MHC class I-presentable peptides and the effect of flanking sequences. J Immunol 154: 1273–1279. 7822796

[81] Eggers M, Boes-Fabian B, Ruppert T, Kloetzel PM, Koszinowski UH (1995) The cleavage preference of the proteasome governs the yield of antigenic peptides. J Exp Med 182: 1865–1870. 7500032 10.1084/jem.182.6.1865 PMC2192259

[82] Del Val M, Schlicht HJ, Ruppert T, Reddehase MJ, Koszinowski UH (1991) Efficient processing of an antigenic sequence for presentation by MHC class I molecules depends on its neighboring residues in the protein. Cell 66: 1145–1153. 1913805 10.1016/0092-8674(91)90037-y

[83] Imai T, Duan X, Hisaeda H, Himeno K (2008) Antigen-specific CD8+ T cells induced by the ubiquitin fusion degradation pathway. Biochem Biophys Res Commun 365: 758–763. 18029260 10.1016/j.bbrc.2007.11.034

[84] Rodriguez F, Whitton JL (2000) Enhancing DNA immunization. Virology 268: 233–238. 10704331 10.1006/viro.2000.0209

[85] Chen JH, Yu YS, Chen XH, Liu HH, Zang GQ, et al (2012) Enhancement of CTLs induced by DCs loaded with ubiquitinated hepatitis B virus core antigen. World J Gastroenterol 18: 1319–1327. 22493545 10.3748/wjg.v18.i12.1319 PMC3319958

[86] Chou B, Hiromatsu K, Okano S, Ishii K, Duan X, et al (2012) Antiangiogenic tumor therapy by DNA vaccine inducing aquaporin-1-specific CTL based on ubiquitin-proteasome system in mice. J Immunol 189: 1618–1626. 22802414 10.4049/jimmunol.1101971

[87] Rezvan H, Rees R, Ali S (2012) Immunogenicity of MHC Class I Peptides Derived from Leishmania mexicana Gp63 in HLA-A2.1 Transgenic (HHDII) and BALB/C Mouse Models. Iran J Parasitol 7: 27–40. 23323089 PMC3537470

[88] Basu R, Roy S, Walden P (2007) HLA class I-restricted T cell epitopes of the kinetoplastid membrane protein-11 presented by Leishmania donovani-infected human macrophages. J Infect Dis 195: 1373–1380. 17397010 10.1086/513439

[89] John L, John GJ, Kholia T (2012) A reverse vaccinology approach for the identification of potential vaccine candidates from Leishmania spp. Appl Biochem Biotechnol 167: 1340–1350. 22434357 10.1007/s12010-012-9649-0

[90] Schroeder J, Aebischer T (2011) Vaccines for leishmaniasis: from proteome to vaccine candidates. Hum Vaccin 7 Suppl: 10–15 10.4161/hv.7.0.14556 21245661

[91] Silverman JM, Reiner NE (2011) Leishmania exosomes deliver preemptive strikes to create an environment permissive for early infection. Frontiers in cellular and infection microbiology 1 10.3389/fcimb.2011.00026 PMC3417360 22919591

[92] Santarém N, Silvestre R, Tavares J, Silva M, Cabral S, et al (2007) Immune response regulation by leishmania secreted and nonsecreted antigens. BioMed Research International 2007 10.1155/2007/85154 PMC1940321 17710243

[93] Abdian N, Gholami E, Zahedifard F, Safaee N, Rafati S (2011) Evaluation of DNA/DNA and prime-boost vaccination using LPG3 against Leishmania major infection in susceptible BALB/c mice and its antigenic properties in human leishmaniasis. Exp Parasitol 127: 627–636. 21187087 10.1016/j.exppara.2010.12.007

[94] Pirdel L, Hosseini AZ, Kazemi B, Rasouli M, Bandehpour M, et al (2012) Cloning and Expression of Leishmania infantum LPG3 Gene by the Lizard Leishmania Expression System. Avicenna J Med Biotechnol 4: 186–192. 23407850 PMC3558223

[95] Descoteaux A, Avila HA, Zhang K, Turco SJ, Beverley SM (2002) Leishmania LPG3 encodes a GRP94 homolog required for phosphoglycan synthesis implicated in parasite virulence but not viability. EMBO J 21: 4458–4469. 12198148 10.1093/emboj/cdf447 PMC126187

[96] Silverman JM, Chan SK, Robinson DP, Dwyer DM, Nandan D, et al (2008) Proteomic analysis of the secretome of Leishmania donovani. Genome Biol 9: R35. 18282296 10.1186/gb-2008-9-2-r35 PMC2374696

