# Supplementary figures and images for: Immunogenicity Evaluation of a Rationally Designed Polytope Construct Encoding HLA-A*0201 Restricted Epitopes Derived from Leishmania major Related Proteins in HLA-A2/DR1 Transgenic Mice: Steps toward Polytope Vaccine
Source: PLoS One. 2014 Oct 13;9(10):e108848. doi: 10.1371/journal.pone.0108848 (PMC4195657; doi:10.1371/journal.pone.0108848)

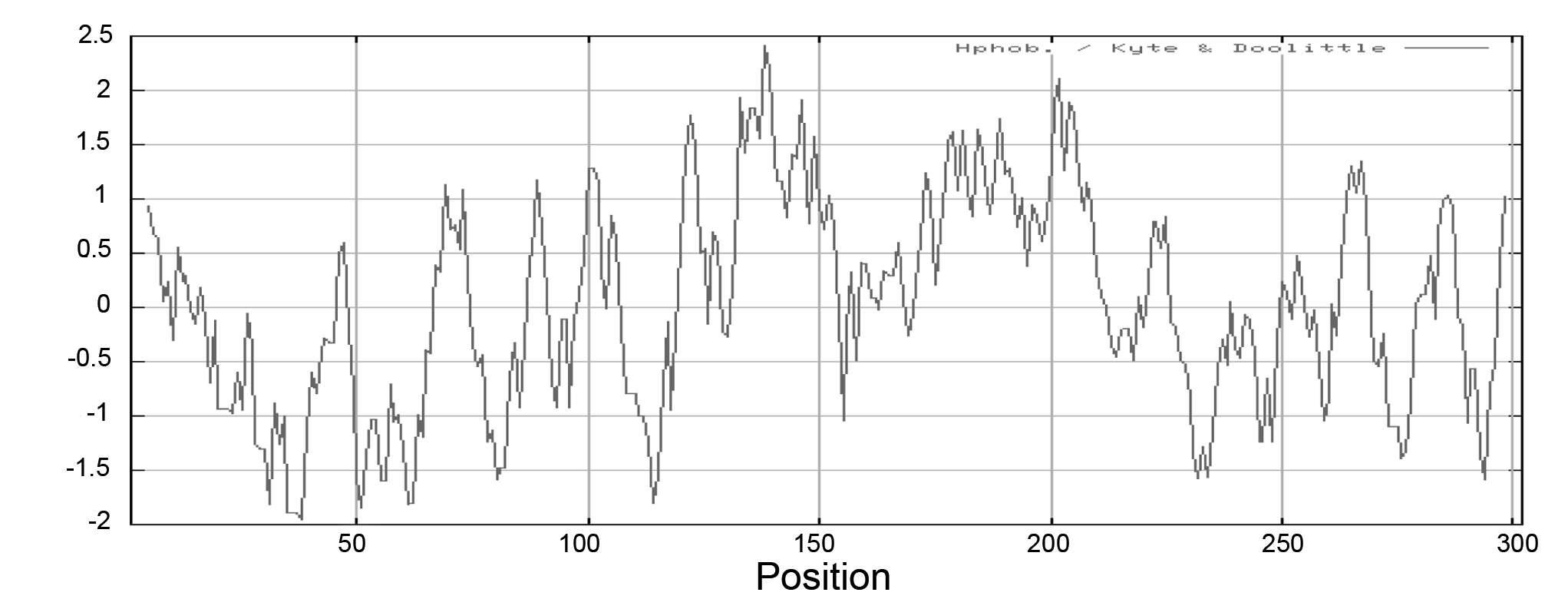

Supplement: Figure S1 — Kate and Dolite analysis (Protscale) of hydrophobic profile of the final polytope arrangement. This aanlysis was used to finally select between different combinations and shows the pattern of the final selected polytope sequence. No hydrophobic patch was detected specially at the N-terminal region to hinder translation. (TIF) [file pone.0108848.s001.tif]

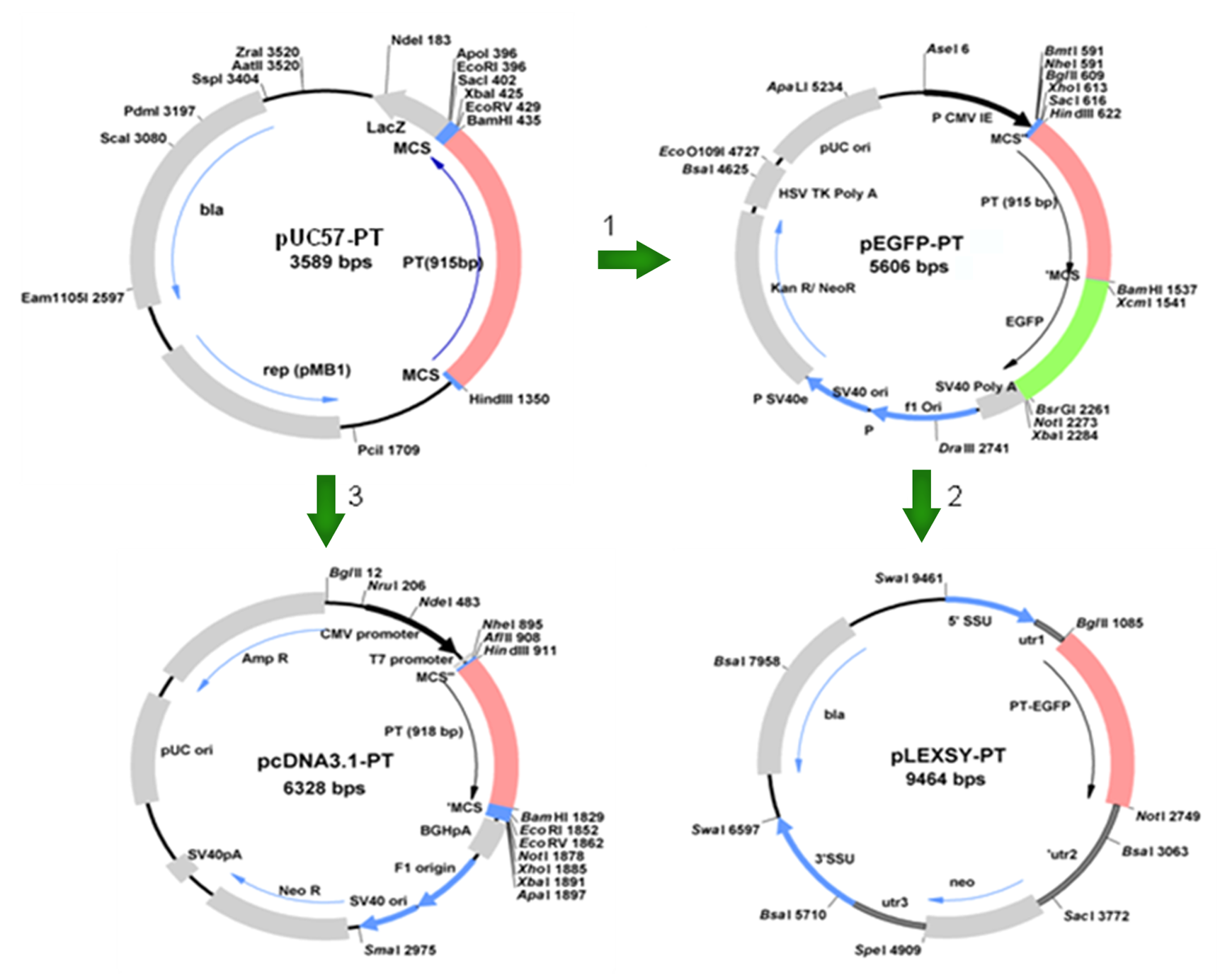

Supplement: Figure S2 — Cloning pathway. 921 bp long polytop (PT) sequence, was codon optimized for optimal expression in mice and received in pUC57 (pUC57-PT). pEGFP-PT was used to confirm the expression of the sequence in mammalian cells by CMV promoter, pLEXSY-PT-EGFP was used to confirm the stability of the expressed polytope and pcDNA-PT was used for inoculations. (TIF) [file pone.0108848.s002.tif]

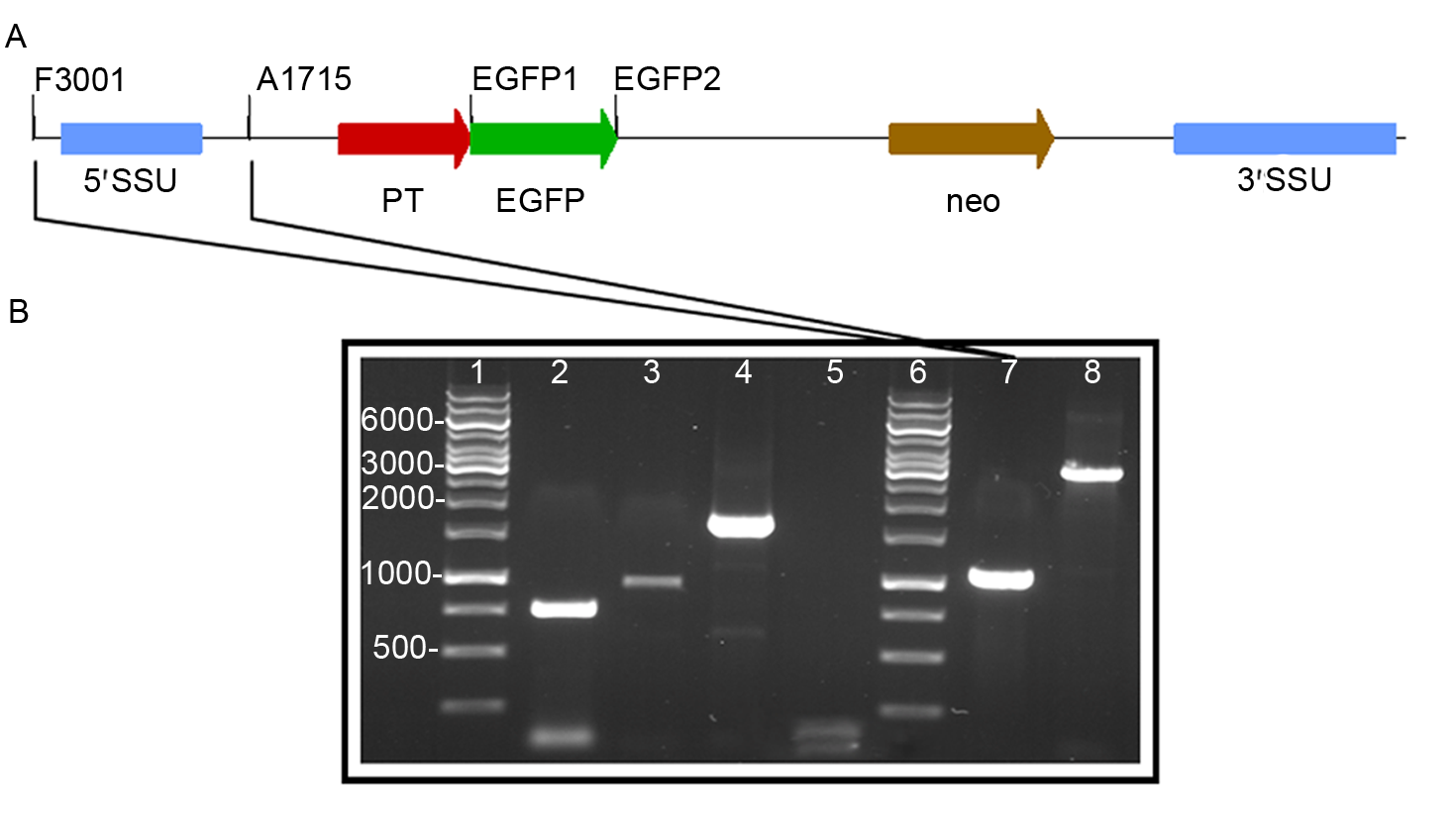

Supplement: Figure S3 — Representative PCR reactions used to confirm the plasmid integration into the genome from one transfected Leishmania tarentolae clone. A. Schematic representation of genome sequence after plasmid integration into rDNA ssu region. B. Full set of PCR reactions followed to confirm the integration at DNA level. Lane 1 and 6: 1 kb DNA ladder marker, lane 2: EGFP fragment (727 bp), lane 3: Polytope fragment (929 bp), lane 4: Polytope-EGFP fragment (1665 bp), lane 7: SSU fragment (1070 bp) and lane 8: EGFP-SSU (3000 bp). Lane 7 points to the most important reaction with 2 primers specific for a chromosomal sequence and plasmid sequence with ssu fragment in between. Lane 5 refers to un-transfected cells confirmed with F3001/A1715 PCR reaction. (TIF) [file pone.0108848.s003.tif]

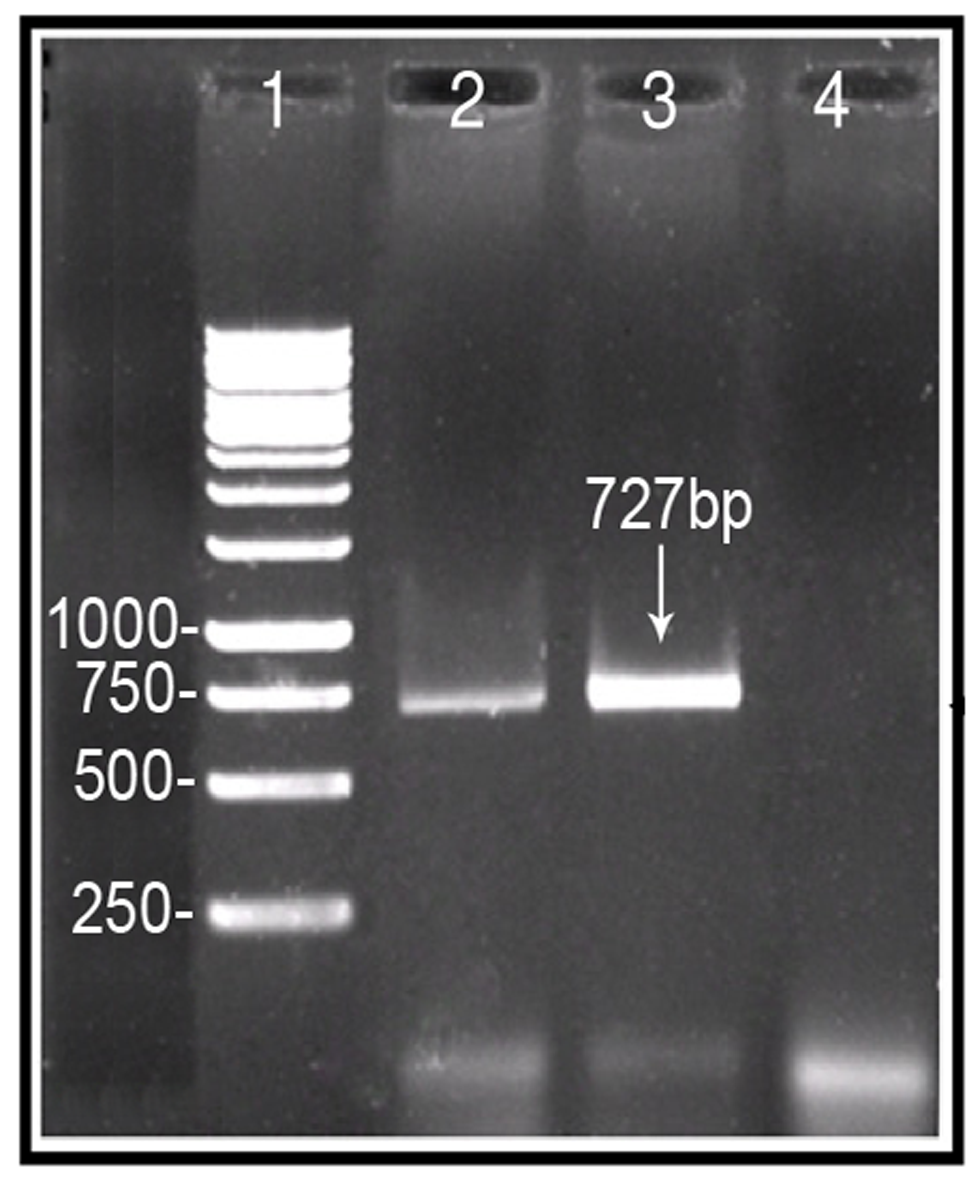

Supplement: Figure S4 — RNA expression evaluation with a set of primers specific for EGFP. Lane 1: Fermentas 1 Kb ladder marker, lane 2 and 3: RT-PCR reaction from 2 transfected clones, lane 3: un-transfected cells. (TIF) [file pone.0108848.s004.tif]

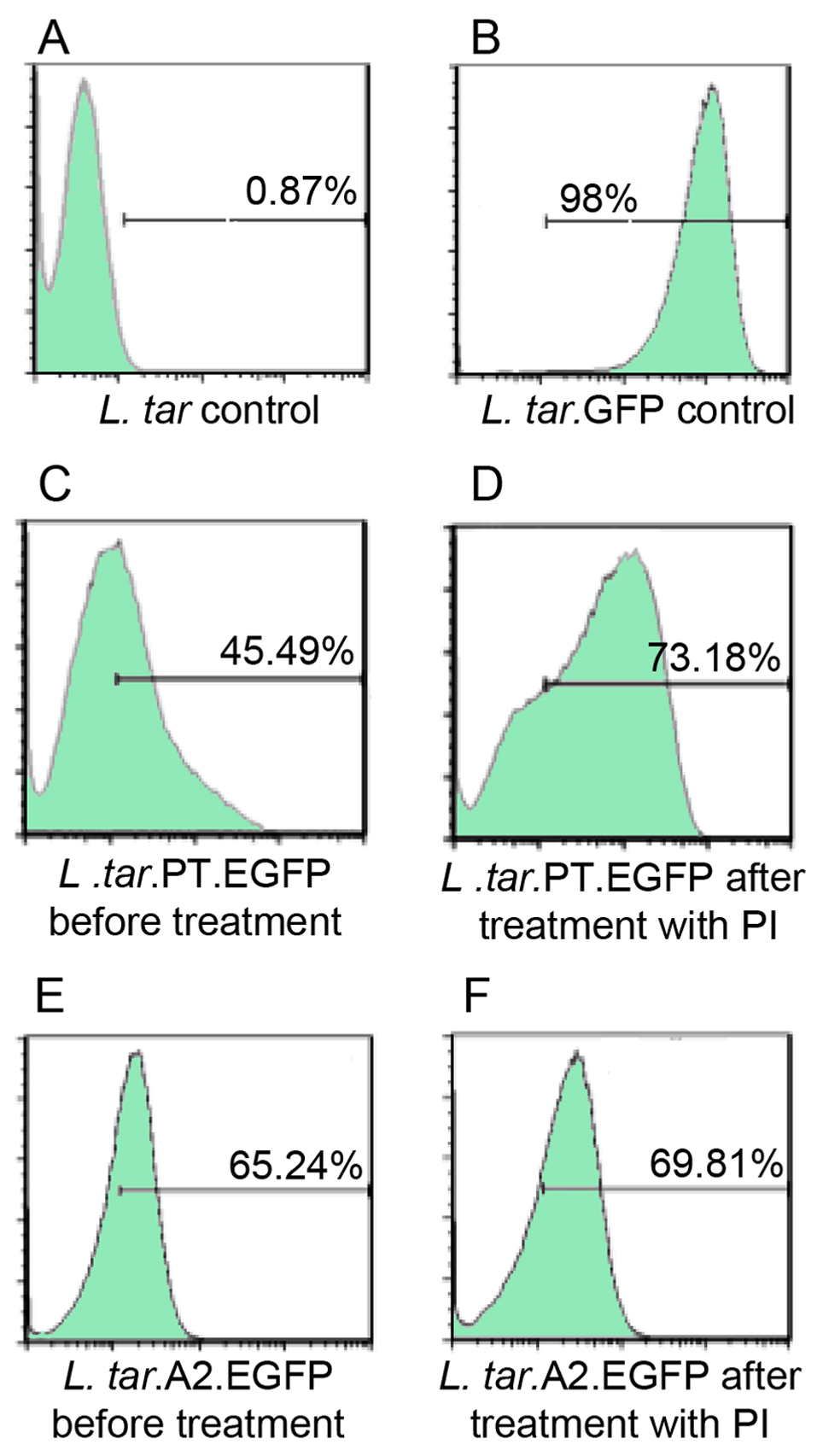

Supplement: Figure S5 — Effect of MG132 treatment on ubiquitinated and non-ubiquitinated constructs. A. un-transfected parasite, B. EGFP transfected parasite, C and D, L. tarentolae transfected with ubiqitinated construct (pLEXSY-PT-EGFP). E and F, L. tarentolae transfected with un-ubiquitinated construct (pLEXSY-A2-EGFP). Expression level of EGFP before and after treatment with proteasome inhibitor roughly differs for un-ubiqitinated protein quite contrary to ubiquitinated protein. Numbers on each plot represent GFP positive population PI: proteasome inhibitor. (TIF) [file pone.0108848.s005.tif]

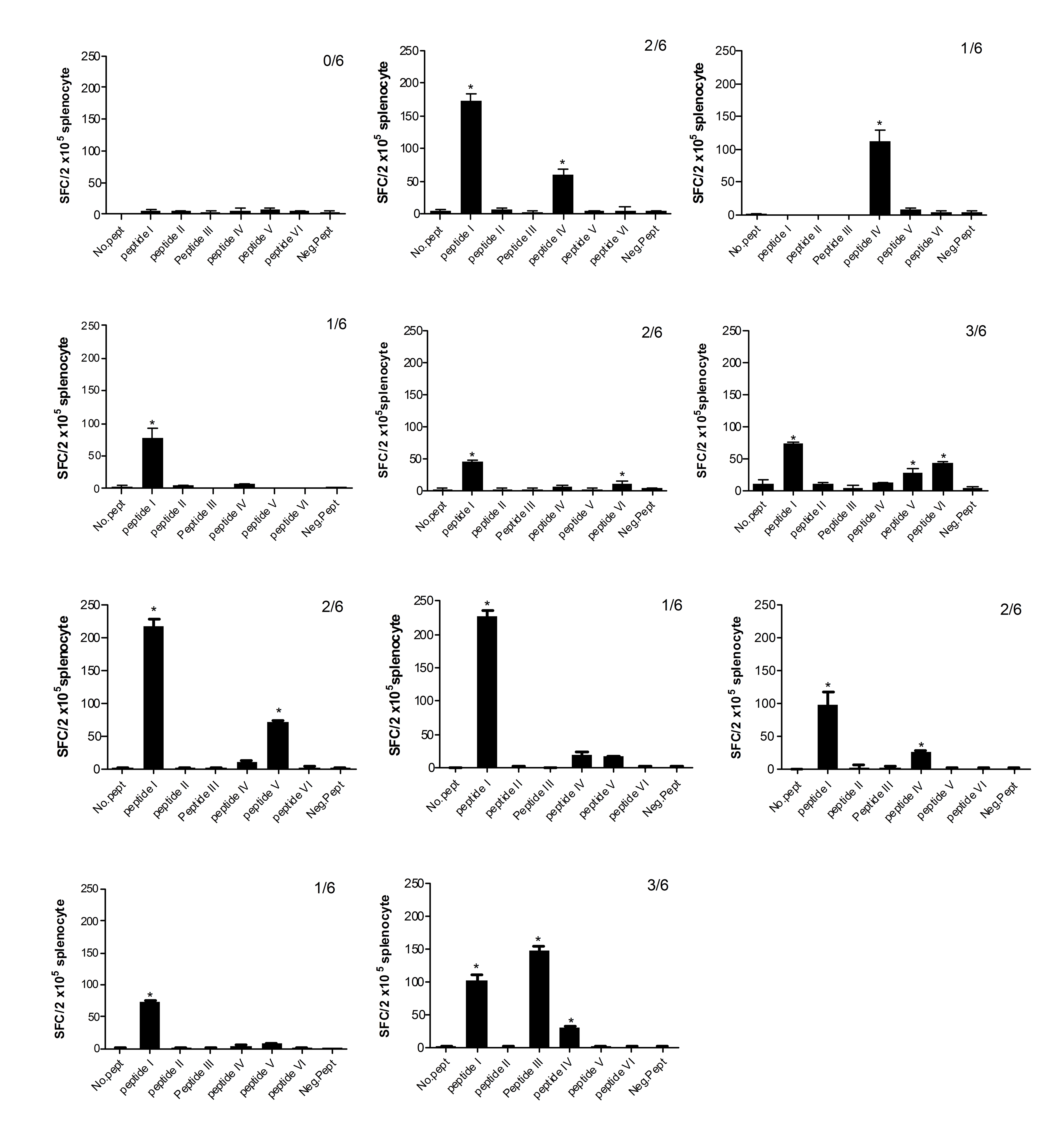

Supplement: Figure S6 — Ex-vivo response of individual mice against six peptides (5 µg/ml/peptide) in HLA A2/DR1 mice. A total of 11 mice in two rounds of experiments were immunized with polytope construct three times with one week interval and sacrificed 10 days after the last booster. Splenocytes from individual mice were in-vitro re-stimulated by representative peptides (P1-P6) of HLA-A2 and specific IFN-γ production was evaluated by ex-vivo ELISPOT assay. Each column represents the mean of duplicate wells stimulated with each peptide. Numbers on each plot show the number of peptides with positive response for each mouse. Peptide stimulations resulting in spots two times the negative control (Neg.pept) and more than 10 were considered positive (stars). Neg.pept (negative control peptide) represents a 9mer HLA-A*0201 restricted peptide from human telomerase. (TIF) [file pone.0108848.s006.tif]

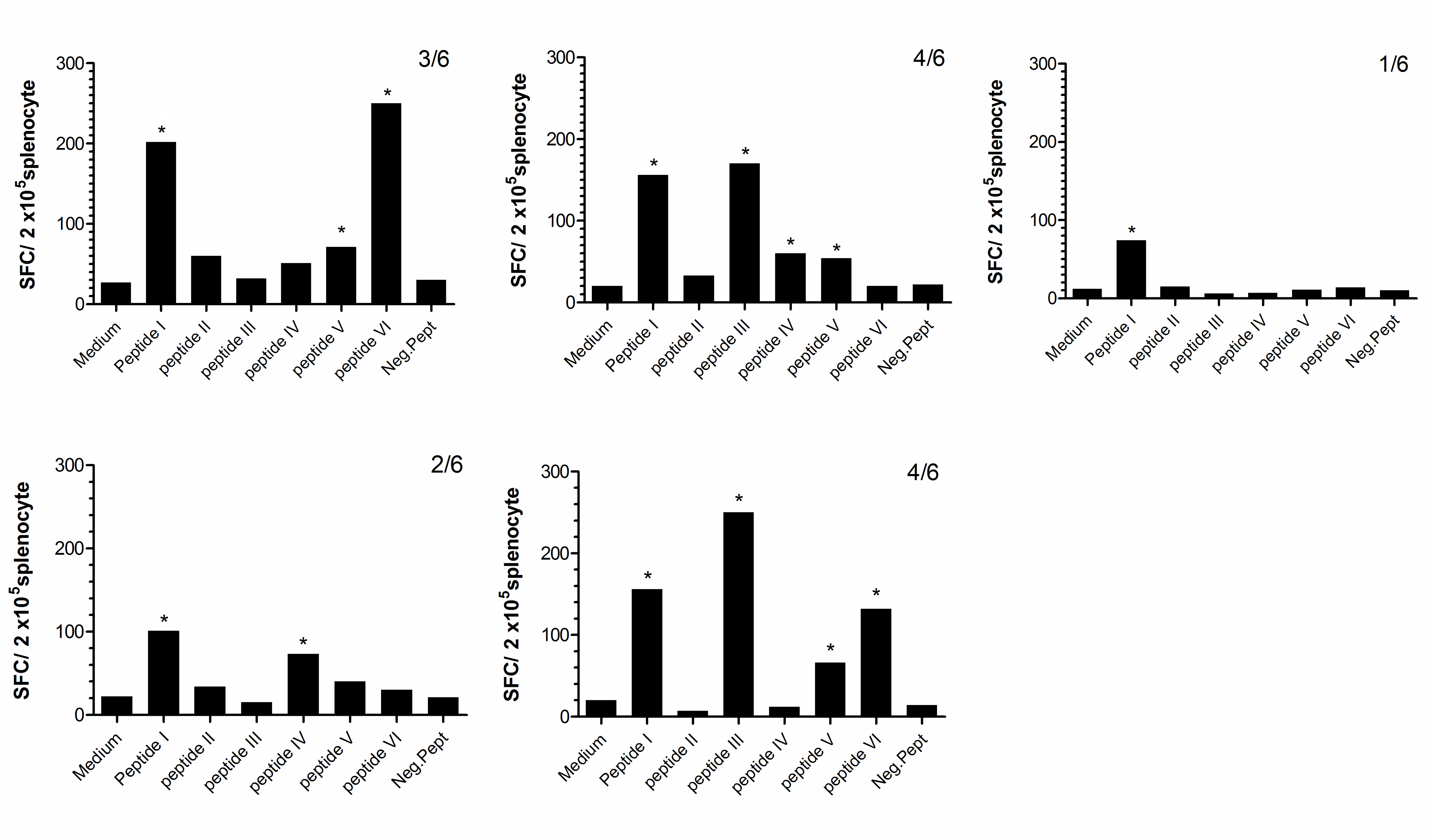

Supplement: Figure S7 — In vitro evaluation of the specific response against six peptides in HLA A2/DR1 mice after one week stimulation (with 100 u/ml IL-2). Splenocytes from a total of 5 mice immunized with polytope construct three times with one week interval and sacrificed 10 days after the last booster were re-stimulated by representative peptides (5 µg/ml/peptide) of HLA-A2. Specific IFN-γ production was evaluated by ex-vivo ELISPOT assay. Each column represents mean of duplicate wells for each individual mice response against each peptide. Numbers on each plot show the number of peptides with positive response for each mouse. Peptide stimulations resulting in spots two times the negative control (Neg.pept) and more than 10 were considered positive (stars). (TIF) [file pone.0108848.s007.tif]

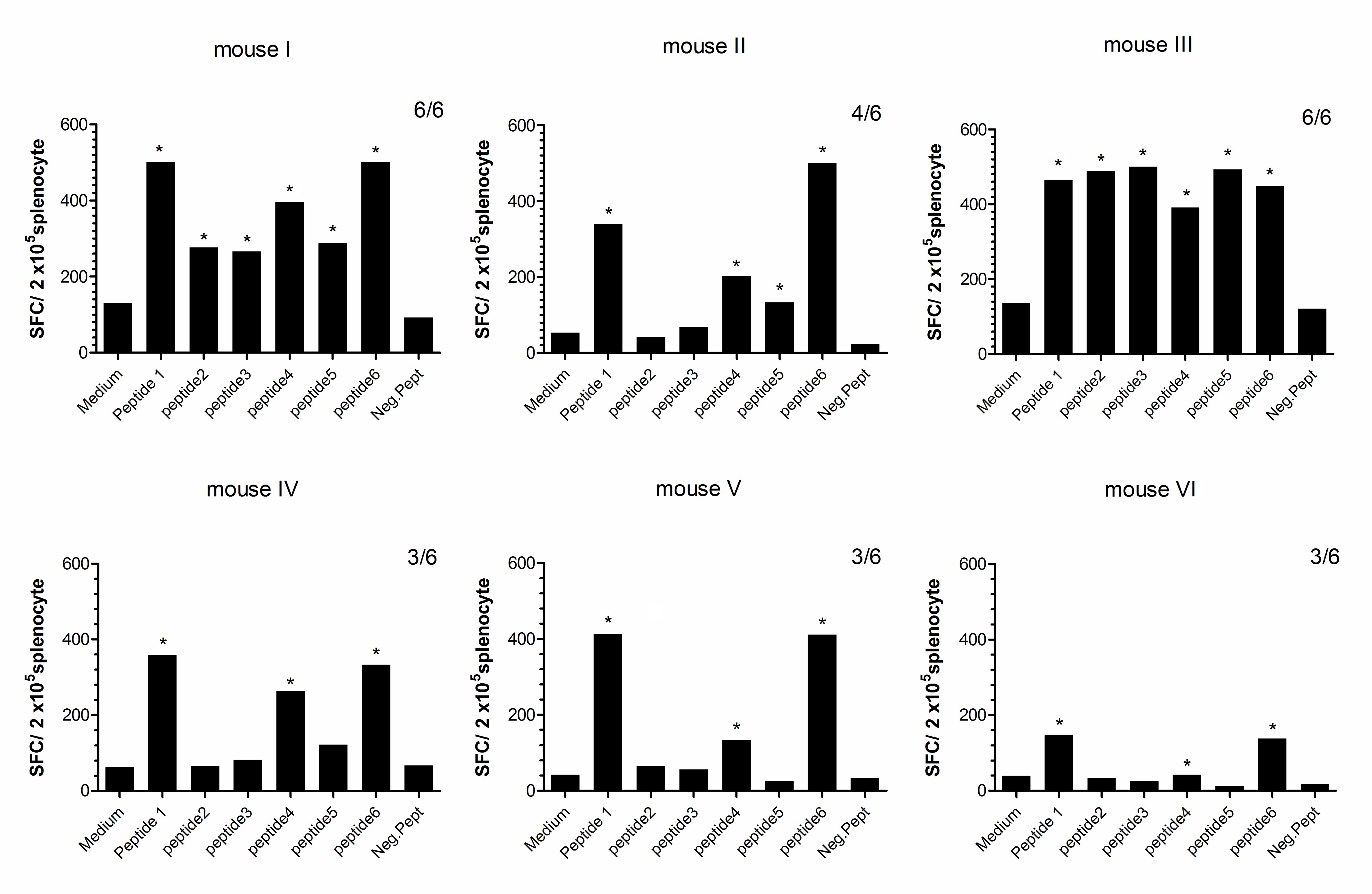

Supplement: Figure S8 — In vitro evaluation of the specific response against six peptides in HLA A2/DR1 mice after one week stimulation with 100 u/ml IL-2 and higher concentration of peptides. Splenocytes were stimulated with 10 µg/ml/peptide instead of 5 µg/ml/peptide. Numbers on each plot show the number of peptides with positive response for each mouse. Peptide stimulations resulting in spots two times the negative control (Neg.pept) and more than 10 were considered positive (stars). (TIF) [file pone.0108848.s008.tif]
